# Unbounded-Time Safety Verification of Guarded LTI Models with Inputs by Abstract Acceleration

**DOI:** 10.1007/s10817-020-09562-z

**Published:** 2020-05-29

**Authors:** Dario Cattaruzza, Alessandro Abate, Peter Schrammel, Daniel Kroening

**Affiliations:** 1 Department of Computer Science, University of Oxford, Oxford, UK; 2 School of Engineering and Informatics, University of Sussex, Brighton, UK

**Keywords:** Safety analysis, Invariant generation, Reachability computation, LTI models, Dynamical models, Abstract acceleration, CEGAR

## Abstract

Reachability analysis of dynamical models is a relevant problem that has seen much progress in the last decades, however with clear limitations pertaining to the nature of the dynamics and the soundness of the results. This article focuses on sound safety verification of unbounded-time (infinite-horizon) linear time-invariant (LTI) models with inputs using reachability analysis. We achieve this using counterexample-guided Abstract Acceleration: this approach over-approximates the reachability tube of the LTI model over an unbounded time horizon by using abstraction, possibly finding concrete counterexamples for refinement based on the given safety specification. The technique is applied to a number of LTI models and the results show robust performance when compared to state-of-the-art tools.

## Introduction

Linear loops are a ubiquitous programming pattern [47]. Linear loops iterate over continuous variables (in the case of physical systems) or discrete variables (in the case of loops in digital programs), which are updated using a linear transformation. Linear loops may be guarded, i.e., halt if a given linear condition holds: at this point the system may either elicit a new behaviour, or simply terminate. Inputs from the environment can be modelled by means of non-deterministic choices within the loop. These features make linear loops expressive enough to capture the dynamics of many hybrid dynamical models [6, 47]. The usage of such models in safety-critical embedded systems makes linear loops a fundamental target for formal methods.

Many high-level requirements for embedded control systems can be modelled as safety properties, i.e., deciding reachability of certain *bad states*, for which the model exhibits unsafe behaviour. Sets of bad states may, in linear loops, be described by means of guard assertions, namely (linear) constraints over their continuous variables. Reachability in linear programs, however, is a formidable challenge for automatic analysers: despite the restriction to linear transformations (i.e., linear dynamics) and linear guards, it is undecidable in the general case. The problem has been related to the Skolem Problem, which includes a subset of cases that are known to be decidable (e.g., the orbit problem [46], and for low-order models [54]). The problem is decidable when restricted to finite state spaces, but even then, algorithms are costly (for the infinite-time case in particular).

The goal of this article is to push the frontiers of unbounded-time reachability analysis: we aim to devise a method that performs sound reasoning about unbounded trajectories by *abstract acceleration*. Abstract acceleration [37, 38, 44] approximates the effect of an arbitrary number of loop iterations (up to infinity) with a single, non-iterative transfer function that is applied to the entry state of the loop (i.e., to the set of initial conditions of the linear dynamics). This article extends the work in [44] to models with non-deterministic inputs, elaborating early work in [57] and completing [14, 16].

The key original contributions of this article are as follows:

1. We present a new technique to include time-varying non-deterministic inputs in the abstract acceleration of general linear loops.
2. We extend abstract acceleration to the continuous time case.
3. We introduce a technique to help the analysis of support functions in complex spaces, in order to increase the precision of previous abstract acceleration methods.
4. We develop a counterexample-guided refinement for Abstract Acceleration for safety verification, maximising speed when high precision is not necessary, thus allowing for optimal analysis within a safe region.
5. We provide an implementation of the discussed procedures as a tool, called *Axelerator*, which is available at http://www.cprover.org/LTI/ We test these novel procedures using a broad set of experiments: our benchmarks (including specifications of the initial states, input ranges and guard sets), are also available.

Finally, in Sect. 10 we provide a thorough review and comparison with related work.

## Preliminaries

### Linear Loops with Inputs—Syntax

A discrete time LTI model may be described as a simple linear loop. Simple linear loops are functions expressed in the form:

$$\begin{aligned} while (\varvec{G}\varvec{x} \le \varvec{h})\ \ \varvec{x} := \varvec{A}\varvec{x}+\varvec{B}\varvec{u}, \end{aligned}$$

where $\varvec{x} \in \mathbb {R}^p$ is a valuation on the state variables, $\varvec{G}\varvec{x} \le \varvec{h}$ is a linear constraint on the states (with $\varvec{G} \in \mathbb {R}^{r \times p}$ and $\varvec{h} \in \mathbb {R}^r$), $\varvec{u} \in \mathbb {R}^q$ is a non-deterministic input, and $\varvec{A} \in \mathbb {R}^{p \times p}$ and $\varvec{B} \in \mathbb {R}^{p \times q}$ are linear transformations characterising the dynamics of the model. This syntax can be interpreted as the dynamics of a discrete-time LTI model with inputs, under the presence of a guard set which, for ease of notation, we denote as $G =\{\varvec{x} : \varvec{G}\varvec{x}\le \varvec{h}\}$. The purpose of the guard is to restrict the dynamics to a given set, either to ensure safety (think for example of speed limits) and/or to change the behaviour of the model under certain conditions (e.g., once we have reached a certain state we begin a new process).

In particular, the special case where $G = \top$ (i.e., “while true”) represents a time-unbounded loop with no guards, for which the discovery of a suitable invariant is paramount.

### Model Semantics

The traces of the model starting from an initial set $X_0\subseteq \mathbb {R}^p$, with inputs restricted to the set $U \subseteq \mathbb {R}^q$, are sequences $\varvec{x}_0 \xrightarrow {\varvec{u}_0} \varvec{x}_1 \xrightarrow {\varvec{u}_1} \varvec{x}_2 \xrightarrow {\varvec{u}_2} \ldots$, where $\varvec{x}_0 \in X_0$ and $\forall k\ge 0, \varvec{x}_{k+1} = \tau (\varvec{x}_k,\varvec{u}_k)$ and $\varvec{u}_k \in U$, satisfying:

1 $$\begin{aligned} \tau (\varvec{x}_k,\varvec{u}_k) = \left\{ \varvec{A}\varvec{x}_k + \varvec{B}\varvec{u}_k \text { given } \varvec{G}\varvec{x}_k \le \varvec{h} \right\} . \; \end{aligned}$$

We extend the point-wise notation above to convex sets of states and inputs ($X_k$ and *U*), and denote the set of states reached from set $X_k$ by $\tau$ in one step as:

2 $$\begin{aligned} \tau (X_k,U)=\left\{ \tau (\varvec{x}_k,\varvec{u}_k) : \varvec{x}_k \in X_k, \varvec{u}_k \in U \right\} . \end{aligned}$$

We furthermore denote the set of states reached from $X_0$ via $\tau$ in *n* steps (*n-reach set*, constrained by *G*), for $n\ge 0$:

3 $$\begin{aligned} \tau ^0(X_0,U) = X_0,\quad \tau ^n(X_0,U) = \tau (\tau ^{n-1}(X_0,U) \cap G,U). \end{aligned}$$

Since the sets $X_0$ and *U* are convex, the transformations $\varvec{A}$ and $\varvec{B}$ are linear, and vector sums preserve convexity, the sets $X_n = \tau ^n(X_0,U)$ are also convex.

We define the *n-reach tube*

4 $$\begin{aligned} {\hat{X}}_n={\hat{\tau }}^n(X_0,U)=\bigcup _{k\in [0,\ldots ,n]} \tau ^k(X_0,U) \end{aligned}$$

as the union of *k*-reach sets over *n* iterations. Moreover, ${\hat{X}} =\bigcup _{n\ge 0} \tau ^n(X_0,U)$ extends the previous notion to an unbounded time horizon (transitive closure).

### Spectral Eigendecomposition

Eigendecomposition [52] denotes the factorisation of a matrix into a canonical form that is characterised by having the non-zero elements (the eigenvalues) only on the main diagonal (note that not all matrices can be factorised this way, as discussed later). Let $\varvec{A} \in \mathbb {R}^p$ be a diagonalizable square matrix. The eigendecomposition yields the equation $\varvec{A}=\varvec{S}\varvec{\varLambda }\varvec{S}^{-1}$, where $\lambda _i=\varvec{\varLambda }_{ii}$ are the eigenvalues of $\varvec{A}$ and $\varvec{S}_{*i}$ their corresponding eigenvectors, sharing the known property $\varvec{A}\varvec{S}_{*i}=\lambda _i\varvec{S}_{*i}$. When a square matrix is not diagonalizable because of repeated eigenvalues, it can be factored into what is known as the Jordan Form, which in addition to the eigenvalues of the matrix, may contain unitary values in the immediate upper diagonal in the case of duplicate eigenvalues: $\varvec{A}=\varvec{S}\varvec{J}\varvec{S}^{-1}$, where:

5 $$\begin{aligned} \varvec{J}= \left[ \begin{array}{lll} \varvec{J}_1 &{}\quad &{}\quad \\ &{}\quad \ddots &{}\quad \\ &{}\quad &{}\quad \varvec{J}_r\\ \end{array} \right] \, \text { and }\, \varvec{J}_{s \in [1,\ldots ,r]}= \left[ \begin{array}{llll} \lambda _s &{}\quad 1 &{}\quad \ldots &{}\quad 0 \\ 0 &{}\quad \lambda _s &{}\quad \ddots &{}\quad \vdots \\ \vdots &{}\quad \ddots &{}\quad \ddots &{}\quad 1 \\ 0 &{}\quad \ldots &{}\quad 0 &{}\quad \lambda _s \\ \end{array} \right] . \end{aligned}$$

In the case of the Jordan Form, the eigenvectors corresponding to repeated eigenvalues are called generalised eigenvectors and have the property that $(\varvec{A}-\lambda _s\varvec{I})^j\varvec{v}_j = 0$, where $\varvec{v}_j$ is the $j{\text {th}}$ generalised eigenvector related to eigenvalue $\lambda _s$.

Finally, the eigenvalues of a real matrix may be complex numbers. This is inconvenient in the subsequent analysis, so rather than using complex arithmetic on these numbers, we choose a different representation over the pseudo-eigenspace. Pseudo-eigendecomposition relies on the observation that complex eigenvalues of a real matrix always come in conjugate pairs. Relaxing the restriction of non-zero values on the main diagonal to include the immediate off-diagonal terms, we leverage the following equivalence:

6 $$\begin{aligned} \begin{array}{ccc} \left[ \begin{array}{cc}\varvec{v}_i&{}\varvec{v}_{i+1}\end{array}\right] &{} \left[ \begin{array}{cc}\lambda _{i}&{}0\\ 0&{}\lambda _{i+1}\end{array}\right]&\left[ \begin{array}{cc}\varvec{v}_i&\varvec{v}_{i+1}\end{array}\right] ^{-1} \end{array}= \begin{array}{ccc} \left[ \begin{array}{cc}\varvec{v}_i&{}\varvec{v}_i^*\end{array}\right] \left[ \begin{array}{cc}re^{\theta i}&{}0\\ 0&{}re^{-\theta i}\end{array}\right] \left[ \begin{array}{cc}\varvec{v}_i&\varvec{v}_i^*\end{array}\right] ^{-1} \end{array} \nonumber \\ =\begin{array}{ccc} \left[ \begin{array}{cc}re(\varvec{v}_i)&{}im(\varvec{v}_i)\end{array}\right] \left[ \begin{array}{cc}r \cos (\theta )&{}r \sin (\theta )\\ -r \sin (\theta )&{}r \cos (\theta )\end{array}\right] \left[ \begin{array}{cc}re(\varvec{v}_i)&im(\varvec{v}_i)\end{array}\right] ^{-1} \end{array}, \end{aligned}$$

where $re(\varvec{v})$ and $im(\varvec{v})$ are the real and imaginary part of $\varvec{v}$, respectively. In the case of a non-diagonal Jordan form, the columns are rearranged first (including the upper diagonal ones), and the conversion above is then performed. This representation is also called the Real Jordan Form.

### Support Functions

#### Definition of Support Functions

**Fig. 1:**
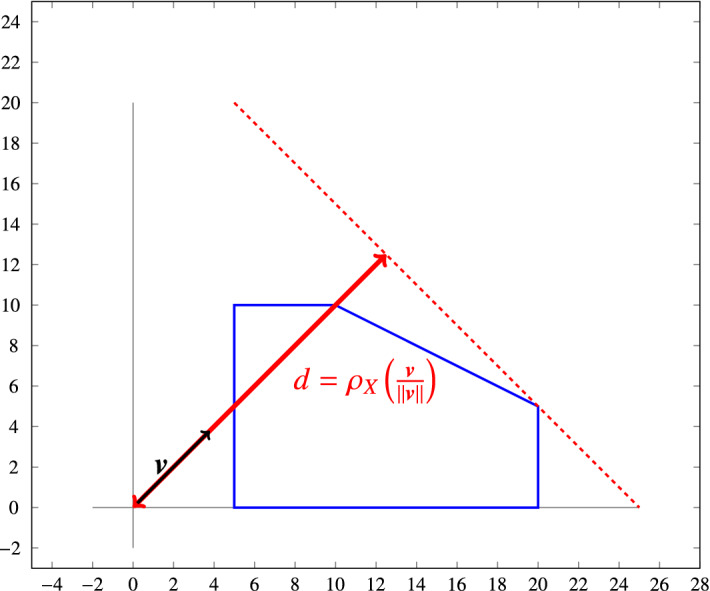
Support function for a polyhedral set in $\mathbb {R}^2$. The distance between the tangent line and the origin is $\rho _X\left( \frac{\varvec{v}}{\Vert \varvec{v} \Vert }\right)$

A support function is a convex function defined over the vector space $\mathbb {R}^p$, which describes the distance of a supporting hyperplane to the origin from a given set in $\mathbb {R}^p$, as illustrated in Fig. 1.

**Fig. 2:**
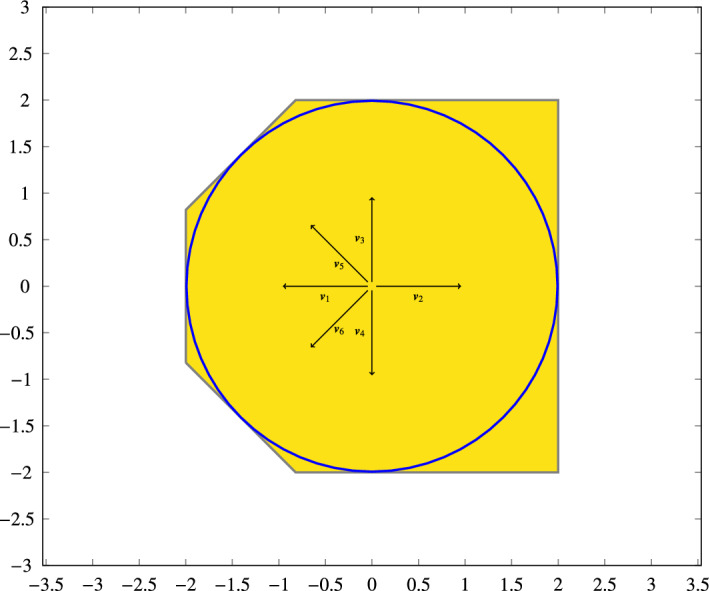
Support function for a circular set using six directions ($\varvec{v}_1 \ldots \varvec{v}_6$). The resulting polyhedron is an over-approximation of the original set (note that directions need not be symmetrical)

Support functions can be used to describe a set by defining the distance of its convex hull with respect to the origin, given a number of directions. More specifically, a support function characterises the distance from the origin to the hyperplane that is orthogonal to the given direction and that touches its convex hull at its farthest. For example, the support function of a sphere centred at the origin given any unit vector $\varvec{v}$ in $\mathbb {R}^3$, evaluates to the radius of the sphere.

The intersection of multiple half spaces, each obtained by sampling a support function in a specific direction, can generate a polyhedron (see Fig. 2), as discussed further in the next section. Finitely sampled support functions (i.e., using a limited number of directions) are template polyhedra in which the directions are not fixed, which helps to avoid wrapping effects (wherein sampling in given directions creates an over-approximation of a set that is not aligned with said directions). The larger the number of distinct directions provided, the more precisely represented the set is. In more detail, given a direction $\varvec{v} \in \mathbb {R}^p$, the support function of a non-empty set $X \subseteq \mathbb {R}^p$ in the direction of $\varvec{v}$ is defined as

$$\begin{aligned} \rho _X : \mathbb {R}^p \rightarrow \mathbb {R}, \quad \rho _X(\varvec{v}) = \sup \{ \varvec{x} \cdot \varvec{v}: \varvec{x} \in X\}, \end{aligned}$$

where $\varvec{x} \cdot \varvec{v}=\sum _{i=0}^p \varvec{x}_i \varvec{v}_i$ is the dot product of the two vectors. Support functions apply to any non-empty set $X \subseteq \mathbb {R}^p$, but they are most useful when representing convex sets. We will restrict ourselves to the use of convex polyhedra, in which case the definition of the support function translates to solving the following linear program:

$$\begin{aligned} \rho _X(\varvec{v}) = \max \{ \varvec{x} \cdot \varvec{v}: \varvec{C}\varvec{x} \le \varvec{d}\} \;. \end{aligned}$$

#### Properties of Support Functions

Several properties of support functions allow us to reduce the complexity of operations. The most significant ones are [34]:

$$\begin{aligned} \begin{array}{lr} \rho _{kX}(\varvec{v}) =\rho _X(k \varvec{v}) = k \rho _X(\varvec{v}) \text { for } k \ge 0,\\ \rho _{AX}(\varvec{v})=\rho _{X}(A^\intercal \varvec{v}) \text { where } A \in \mathbb {R}^{p \times p},\\ \rho _{X_1 \oplus X_2}(\varvec{v})=\rho _{X_1}(\varvec{v})+\rho _{X_2}(\varvec{v}),\\ \rho _X(\varvec{v}_1 + \varvec{v}_2) \le \rho _X({\varvec{v}_1}) + \rho _X({\varvec{v}_2}),\\ \rho _{conv(X_1 \cup X_2)}(\varvec{v}) = \max \{\rho _{X_1}(\varvec{v}),\rho _{X_2}(\varvec{v})\},\;\;\;\;\;\;\\ \rho _{X_1 \cap X_2}(\varvec{v}) \le \min \{\rho _{X_1}(\varvec{v}),\rho _{X_2}(\varvec{v})\},\\ \end{array} \end{aligned}$$

where $\varvec{v},\varvec{v}_1,\varvec{v}_2 \in \mathbb {R}^p$. As can be seen by their structure, some of these properties reduce the complexity to lower-order polynomial or even to constant time, by turning matrix-matrix multiplications (${\mathcal {O}}(p^3)$) into matrix-vector (${\mathcal {O}}(p^2)$), or into scalar (${\mathcal {O}}(p)$) multiplications.

#### Support Functions in Complex Spaces

The literature does not state, to the best of our knowledge, any use of support functions in complex spaces. Since we are applying their concept to eigenspaces, which may have complex conjugate eigenvalues, we extend the definition of support functions to encompass the corresponding operations on complex spaces, which are given explicitly.

##### Theorem 1

A support function in a complex vector field is a transformation:

$$\begin{aligned} \rho _X(\varvec{v}) : \mathbb {C}^p \rightarrow \mathbb {R}= \sup \{re(\varvec{x} \cdot \varvec{v}): \varvec{x} \in X \subseteq \mathbb {C}^p, \varvec{v} \in \mathbb {C}^p\}, \end{aligned}$$

where $re(\cdot )$ defines the real part of a complex number. The dot product used here is commonly defined in a complex space as:

$$\begin{aligned} \varvec{a} \cdot \varvec{b} = \sum _{i=0}^p a_ib_i^*\ , \ \ \ \varvec{a}, \varvec{b} \in \mathbb {C}^p, \end{aligned}$$

where the element $b_i^*$ is the complex conjugate of $b_i$.

##### Proof

Let $$f : \mathbb {C}^p \rightarrow \mathbb {R}^{2p}$$ and

$$\begin{aligned} \varvec{x}' = f(\varvec{x}) = \left[ \begin{array}{c}re(\varvec{x})\\ im(\varvec{x})\end{array}\right] , \quad \varvec{v}' = f(\varvec{v}^*) = \left[ \begin{array}{c}re(\varvec{v})\\ -im(\varvec{v}) \end{array}\right] . \end{aligned}$$

Using abstract interpretation [23] we define a Galois connection $\alpha (\varvec{x})= f(\varvec{x})$ and $\gamma (\varvec{x}')= f^{-1}(\varvec{x}')$, which is clearly a one-to-one relation. We can therefore establish an equivalence between $\rho _X(\varvec{v}) = \rho _{X'}(\varvec{v}')$. $\square$

As we shall see later, the imaginary part of the dot product is not relevant to the support function, and we therefore disregard it. Using properties of support functions, we now have:

$$\begin{aligned} \rho _X(r e^{i\theta } \varvec{v}) = r \rho _X(e^{i\theta }\varvec{v}), \end{aligned}$$

which is consistent with the real case when $\theta =0$. The reason why $e^{i\theta }$ cannot be factored out as a constant is because it executes a rotation on the vector, and therefore follows the same rules as a matrix multiplication, namely:

$$\begin{aligned} \rho _X(e^{i\theta }\varvec{v})&\triangleq \rho _X \left( \varvec{v}_2\right) , \text { where } \\ \left[ \begin{array}{c} re(\varvec{v}_2)\\ im(\varvec{v}_2) \end{array}\right]&= \left[ \begin{array}{ll} \cos \theta &{}\quad -\sin \theta \\ \sin \theta &{}\quad \cos \theta \end{array}\right] \left[ \begin{array}{c} re(\varvec{v})\\ im(\varvec{v}) \end{array}\right] . \end{aligned}$$

Notice the resemblance of this matrix to a pseudo-eigenvalue representation (see Eq. (6)). Since the vectors we are interested in are conjugate pairs (because they have been created by a transformation into a matrix eigenspace), we can transform our problem into a pseudo-eigenvalue representation. Since this removes the imaginary component from the setup, we can evaluate the support function using standard methods and properties, by transforming the conjugate pairs into separate vectors representing the real and imaginary parts and rotation matrices as in the equation above.

Recently, Adimoolam and Dang [1] have presented a calculus for complex zonotopes that exploits the same idea, namely using complex eigenvalues to represent ellipsoidal and linear boundaries for reach sets. Their projections into real space correspond to our semi-spherical approximations for variable inputs. Apart from the basic difference in domain (zonotopes vs. polyhedra), which in itself changes some aspects of the problem, the main difference is that the authors perform their analysis over the complex space, whereas we ultimately apply the method in the real space by using pseudo-eigenspaces.

### Convex Polyhedra

A polyhedron is a subset of $\mathbb {R}^p$ with planar faces. Each face is supported by a hyperplane that creates a half-space, and the intersections of these hyperplanes are the edges (and vertices) of the polyhedron. A polyhedron is said to be convex if a line segment joining any two points of its surface is contained within its interior. Convex polyhedra are better suited than general polyhedra to define an abstract domain, mainly because they have a simpler representation and because operations over convex polyhedra are in general easier than over general polyhedra. There are a number of properties of convex polyhedra that make them ideal for abstract interpretation over continuous spaces, including their ability to reduce an uncountable set of real points into a countable set of faces, edges and vertices. Convex polyhedra retain their convexity across linear transformations, and are functional across a number of operations because they have a dual representation [32], as detailed next. The algorithm to switch between these two representations is given in Sect. 2.6.5.

#### Vertex Representation

Since every edge in the polyhedron corresponds to a line between two vertices, and every face corresponds to the area enclosed by a set of co-planar edges, a full description of the polyhedron is obtained simply by listing its vertices. Since linear operations retain the topological properties of the polyhedron, performing these operations on the vertices is sufficient to obtain a complete description of the transformed polyhedron (defined by the transformed vertices). Formally, a polyhedron can be described as a set $V \in \mathbb {R}^p$ such that $\varvec{v} \in V$ is a vertex of the polyhedron.

#### Inequality Representation (a.k.a. Face Representation)

The dual of the Vertex representation is the inequality representation, where each inequality represents a face of the polyhedron. Each face corresponds to a bounding hyperplane of the polyhedron (with the edges being the intersection of two hyperplanes and the vertices being the intersection of *p* or more hyperplanes), and is described mathematically as a function of the vector that is normal to the hyperplane. This representation can be made minimal by eliminating redundant inequalities that do not correspond to any face of the polyhedron. If we examine this description closely, we can see that it corresponds to the support function of the vector normal to the hyperplane. Given this description we formalise the following: A convex polyhedron is a topological region in $\mathbb {R}^p$ described by the set

7 $$\begin{aligned} X=\{\varvec{x} \in \mathbb {R}^{p}: \varvec{C}\varvec{x} \le \varvec{d},\, \varvec{C} \in \mathbb {R}^{m \times p},\, \varvec{d} \in \mathbb {R}^{m}\}, \end{aligned}$$

where the rows $\varvec{C}_{i,*}$ for $i \in [1,\ldots ,m]$ correspond to the transposed vectors normal to the faces of the polyhedron, and $\varvec{d}_i$ for $i \in [1,\ldots ,m]$ to the value of the support function of X in the corresponding direction. For simplicity in the presentation, we will extend the use of the support function operator as follows:

$$\begin{aligned} \rho '_X&: \mathbb {R}^{m \times p} \rightarrow \mathbb {R}^m,\quad \rho '_X(\varvec{M}) = \left[ \begin{array}{c} \rho _X(\varvec{(\varvec{M}_{1,*})^\intercal )}\\ \rho _X(\varvec{(\varvec{M}_{2,*})^\intercal )}\\ \vdots \\ \rho _X(\varvec{(\varvec{M}_{m,*})^\intercal })\end{array}\right] . \end{aligned}$$

### Operations on Convex Polyhedra

There are a number of operations that we need to be able to perform on convex polyhedra.

#### Translations

Given a vertex representation *V* and a translation vector $\varvec{t}$, the transformed polyhedron is:

$$\begin{aligned} V'=\{ \varvec{v}+\varvec{t} : \varvec{v} \in V \}\,. \end{aligned}$$

Given an inequality representation *X* and a translation vector $\varvec{t}$, the transformed polyhedron is:

$$\begin{aligned} X'= \left\{ \varvec{x} : \varvec{C}\varvec{x} \le \varvec{d}+\varvec{C}\varvec{t}\right\} . \end{aligned}$$

#### Linear Transformations

Given a vertex representation *V* and a linear transformation $\varvec{L}$, the transformed polyhedron is:

$$\begin{aligned} V'=\varvec{L}V \,. \end{aligned}$$

Given an inequality representation *X* and a linear transformation $\varvec{L}$, the transformed polyhedron corresponds to

$$\begin{aligned} X'\subseteq \left\{ \varvec{x} : \varvec{C}\varvec{L}^+\varvec{x} \le \rho '_X((\varvec{L}^+)^\intercal \varvec{C}^\intercal )\right\} , \end{aligned}$$

where $\varvec{L}^+$ represents the pseudo-inverse of $\varvec{L}$ [55]. In the case when the inverse $\varvec{L}^{-1}$ exists, then:

$$\begin{aligned} X'=\left\{ \varvec{x} : \varvec{C}\varvec{L}^{-1}\varvec{x} \le \varvec{d}\right\} . \end{aligned}$$

From this we can conclude that linear transformations are more efficient when using vertex representation, except when the inverse of the transformation exists and is known a priori. This work makes use of this assumption to avoid alternating between representations.

#### Set Sums

The addition of two polyhedra is defined such that the resulting set contains the sum of all pairs of points inside the original polyhedra. This operation is commonly known as the *Minkowski sum*, namely:

$$\begin{aligned} A \oplus B = \{a + b \,|\, a\in A, b\in B\}. \end{aligned}$$

Given two vertex representations $V_1$ and $V_2$, the resulting polyhedron is

$$\begin{aligned} V=conv(V_1 \oplus V_2), \end{aligned}$$

where $conv(\cdot )$ is the convex hull of the set of vertices contained in the Minkowski sum. Let

$$\begin{aligned} X_1 = \{ \varvec{x} : \varvec{C}_1\varvec{x} \le \varvec{d}_1 \}, \quad X_2 = \{ \varvec{x} : \varvec{C}_2\varvec{x} \le \varvec{d}_2 \}, \quad \end{aligned}$$

be two sets, then

$$\begin{aligned} X_1 \oplus X_2 \subseteq X = \{ \varvec{x} : \varvec{C}\varvec{x} \le \varvec{d} \} , \end{aligned}$$

where

$$\begin{aligned} \varvec{C}=\left[ \begin{array}{c}\varvec{C}_1\\ \varvec{C}_2\end{array}\right] ,\ \varvec{d} = \left[ \begin{array}{c}\varvec{d}_1+\rho '_{X_2}(\varvec{C}_1^\intercal )\\ \varvec{d}_2+\rho '_{X_1}(\varvec{C}_2^\intercal )\end{array}\right] . \end{aligned}$$

Because these sets correspond to systems of inequalities, they can be reduced by removing redundant constraints. Note that if $\varvec{C}_1=\varvec{C}_2$, then

$$\begin{aligned} X&=X_1 \oplus X_2 = \{ \varvec{x} : \varvec{C}_1\varvec{x} \le \varvec{d}_1+\varvec{d}_2 \}. \end{aligned}$$

#### Set Hadamard Products

##### Definition 1

Given two vertex representations $V'$ and $V''$, we define the set Hadamard product operation using such representations as

$$\begin{aligned} V=V' \circ V'' =conv(\{ \varvec{v}' \circ \varvec{v}'' : \varvec{v}' \in V', \varvec{v}'' \in V'' \}), \end{aligned}$$

where $\circ$ represents the Hadamard (coefficient-wise) product of the vectors.

##### Lemma 1

The set $V=V' \circ V''$ introduced in the previous definition is a convex set that contains all possible combinations of products between elements of sets $V'$ and $V''$.

##### Proof

Given a convex set *X* with a vertex representation *V*, by definition we have

$$\begin{aligned} X'=\left\{ t \varvec{v}_1 + (1-t) \varvec{v}_2, \varvec{v}_1,\varvec{v}_2 \in V, t \in [0, 1] \right\} \subseteq X, \end{aligned}$$

which extends to multiple points [13] as

$$\begin{aligned} X'=\left\{ \sum _{i=1}^{m} k_i\varvec{v}_i, \sum _{i=1}^{m} k_i =1, \varvec{v}_i \in V, \text { and } m=|V| \right\} \subseteq X. \end{aligned}$$

Applying the Hadamard product, we obtain

$$\begin{aligned} X'= \{\varvec{x}_1 \circ \varvec{x}_2 : \varvec{x}_1 \in X_1, \varvec{x}_2 \in X_2 \} \subseteq X= X_1 \circ X_2, \end{aligned}$$

where $x_1 \circ x_2 = \sum _{i=1}^{|V'|} k_i\varvec{v}_i' \circ \sum _{j=1}^{|V''|} k_j\varvec{v}_j'' \text { with } \varvec{v}_i' \in V', \varvec{v}_j'' \in V''$. Simplifying, we obtain

$$\begin{aligned} x_1 \circ x_2 =&\sum _{i=1}^{|V'|}\sum _{j=1}^{|V''|} k_i\varvec{v}_i' \circ k_j\varvec{v}_j'' =\sum _{i=1}^{|V'|}\sum _{j=1}^{|V''|} k_ik_j\varvec{v}_i' \circ \varvec{v}_j'' =\sum _{ij=1}^{|V'||V''|}k_{ij}\varvec{v}_{ij}, \end{aligned}$$

where $$\varvec{v}_{ij}=\varvec{v}_i' \circ \varvec{v}_j'' \in V \text { and } \sum _{ij=1}^{|V'||V''|}k_{ij}=\sum _{i=1}^{|V'|}\sum _{j=1}^{|V''|} k_ik_j = 1$$. $$\square $$

Note that in the case of the inequality representation, there is no direct result for this product. We therefore enumerate the sets in one of the polyhedra, and use linear solving algorithms to find an over-approximation:

8 $$\begin{aligned} X \subseteq \left\{ \varvec{x} \,|\, \varvec{x} \cdot \varvec{t} < \max \{\,\rho _{X_2}(\varvec{t}\circ \varvec{v}') \mid \varvec{v}' \in V'\},\, \varvec{t} \in T \right\} , \end{aligned}$$

where $\varvec{t}$ is a template direction for a face in the over-approximation, T is the set of directions selected for the over-approximation, and $V'$ is the set of vertices of *X*.

#### Vertex Enumeration

The vertex enumeration algorithm obtains a list of all vertices of a polyhedron, given a face representation of its bounding hyperplanes. Given the duality of the problem, it is also possible to find the bounding hyperplanes given a vertex description if the chosen algorithm exploits this duality. In this case the description of V is given in the form of a matrix inequality $$\varvec{V}\varvec{x} \le [\begin{array}{cccc}1&1&\cdots&1\end{array}]^\intercal $$ with $$\varvec{V}=[\begin{array}{ccc}\varvec{v}_1&\cdots&\varvec{v}_m\end{array}]^\intercal , \varvec{v}_i \in V$$. Similarly, $\varvec{C}$ can be described as a set containing each of its rows. There are two algorithms that efficiently solve the vertex enumeration problem. lrs [4] is a reverse search algorithm, while cdd [32] follows the double description method. In this work we use the cdd algorithm for convenience in implementation (the original cdd was developed for floats, whereas lrs uses rationals). The techniques presented here can be applied to either. Let

$$\begin{aligned} {\mathcal {C}} = \{ \varvec{x}: \varvec{C}\varvec{x} \ge 0, \varvec{C} \in \mathbb {R}^{n \times p}, \varvec{x} \in \mathbb {R}^p\}. \end{aligned}$$

be the polyhedral cone represented by $\varvec{C}$. The pair $(\varvec{C},V)$ is said to be a double description pair if

$$\begin{aligned} {\mathcal {C}}=\left\{ \varvec{\lambda }^\intercal V : V \in \mathbb {R}^p, \varvec{\lambda } \in \mathbb {R}_{\ge 0}^{|V|}\right\} , \end{aligned}$$

where *V* is called the *generator* of *X*. Each element in *V* lies in the cone of *X*, and its minimal form (smallest *m*) has a one-to-one correspondence with the extreme rays of *X* if the cone is pointed (i.e., it has a vertex at the origin). This last criterion can be ensured by translating a polyhedral description so that it includes the origin, and then translating the vertices back once they have been discovered (see Sect. 2.6).

We also note that:

$$\begin{aligned}&\left\{ \varvec{x} : \varvec{C}\varvec{x} \le \varvec{d}\right\} =\left\{ \varvec{x}' : [\begin{array}{cc}-\varvec{C}&\varvec{d}\end{array}]\,\varvec{x}' \ge 0\right\} ,\;\; \text { where }\varvec{x} \in \mathbb {R}^p\text { and }\varvec{x}'= \left[ \begin{array}{c}\varvec{x}\\ 1\end{array}\right] \in \mathbb {R}^{p+1}. \end{aligned}$$

The vertex enumeration algorithm starts by finding a base ${\mathcal {C}}_K$ which contains a number of vertices of the polyhedron. This can be done by pivoting over a number of different rows in $\varvec{C}$ and selecting the feasible visited points, which are known to be vertices of the polyhedron (pivoting *p* times will ensure at least one vertex is visited if the polyhedron is non-empty). ${\mathcal {C}}_K$ is represented by $\varvec{C}_K$ which contains the rows used for the pivots. The base ${\mathcal {C}}_K$ is then iteratively expanded to ${\mathcal {C}}_{K+i}$ by exploring the $i{\text {th}}$ row of $\varvec{C}$ until ${\mathcal {C}}_K= {\mathcal {C}}$. The corresponding pairs $(\varvec{C}_{K+i},V_{K+i})$ are constructed using the information from $(\varvec{C}_K,V_K)$ as follows.

Let $$\varvec{C}_K \in \mathbb {R}^{n_K \times p}$$, $$\varvec{C}_{i,*} \in \mathbb {R}^{1 \times p}$$, $$V_K \in \mathbb {R}^p$$,

$$\begin{aligned} H_i^+{=}\{\varvec{x} : \varvec{C}_{i,*}\varvec{x} >0 \}, \quad H_i^-=\{\varvec{x} : \varvec{C}_{i,*}\varvec{x} <0 \}, \quad \text { and } \quad H_i^0{=}\{\varvec{x} : \varvec{C}_{i,*}\varvec{x} =0 \}, \end{aligned}$$

be the spaces outside, inside and on the $i{\text {th}}$ hyperplane and

$$\begin{aligned} V_K^+=\{\varvec{v}_j \in H_i^+\}, \quad V_K^-=\{\varvec{v}_j \in H_i^-\}, \quad \text { and } \quad V_K^0=\{\varvec{v}_j \in H_i^0\}, \end{aligned}$$

the existing vertices lying on each of these spaces. Then [32],

$$\begin{aligned} V_{K+i}=V_K^+ \cup V_K^- \cup V_K^0 \cup V_K^i,\quad V_K^i=\left\{ (\varvec{C}_{i,*}\varvec{v}^+)\varvec{v}^--(\varvec{C}_{i,*}\varvec{v}^-)\varvec{v}^+ \mid \varvec{v}^- \in V^-, \varvec{v}^+ \in V^+ \right\} . \end{aligned}$$

## Abstract Acceleration—Overview of the Algorithm

Abstract acceleration is a method that seeks to precisely describe the dynamics of a transition system over a number of steps using a concise description between the first and final steps. More precisely, it looks for a direct formula to express the post-image of an unfolded loop from its initial states. Formally, given the dynamics in Eq. (1), an acceleration formula aims to compute the reach tube based on (3) using a function *f* such that $f(\cdot )=\tau ^n(\cdot )$. In the case of models without inputs, this equation can be derived from the expression $\varvec{x}_n=\varvec{A}^n\varvec{x}_0$.

### Overview of the Algorithm

The basic steps required to compute a reach tube using abstract acceleration are given in Fig. 3.

1. The process starts by performing eigendecomposition of the dynamics (based on matrix $\varvec{A}$) in order to transform the problem into a simpler one. Since we use unsound arithmetic at this stage, the results are quantities that are marked using a tilde (as in $\tilde{\varvec{S}}, \tilde{\varvec{J}}$).
2. The second step involves upper-bounding the rounding errors in order to obtain sound results: bounds on eigenvalues, for example, are well known from the literature and can be obtained as $|\lambda - {\tilde{\lambda }}| < \Vert \tilde{\varvec{S}}\tilde{\varvec{J}}\tilde{\varvec{S}}^{-1}-\varvec{A} \Vert _2$. In general, a variety of off-the-shelf tools may be used, but since larger problems require numerical algorithms for scalability, all subsequent steps are performed using interval arithmetic in order to maintain soundness: we identify corresponding interval-based quantities with bold symbols (e.g., ${\mathbb {S}}, {\mathbb {J}}$), as well as subsequent matrix operations (e.g., computing the inverse of ${\mathbb {S}}$). Thus we obtain $\Vert {\mathbb {S}}{\mathbb {J}}{\mathbb {S}}^{-1}-\mathbb {A} \Vert _2$ by extending the original unsound matrices by one least-significant bit element-wise. We also note that this equation is symmetric, which is why we use the estimated eigenvectors to calculate the error on the original eigenvalues (see [16] for further details). While bounds on the eigenvectors can be calculated from the eigenvalues, we choose a more complex yet faster over-approximation, which is described in [16].
3. The inverse of the generalised eigenvectors ($\mathbb {S}^{-1}$) is calculated soundly (using interval arithmetic).
4. The problem is transformed into canonical form by multiplying both sides of the equation by $\mathbb {S}^{-1}$ (we use blackboard symbols to indicate interval vectors and matrices, which are employed to ensure sound operations), obtaining
  $$\begin{aligned} X_k' {=}\mathbb {J}\left( X_{k-1}' \cap G' \right) +U', \quad \text { where } \quad X_k' {=}\mathbb {S}^{-1}X_k, \quad U'{=}\mathbb {S}^{-1}\varvec{B}U,\quad G'{=}\{\varvec{x} \mid \varvec{G}\mathbb {S}\varvec{x} \le \varvec{h} \}. \end{aligned}$$
5. We calculate the number of iterations *n* based on the guard set, as explained in Sect. 7. If there are no guards, we set $n=\infty$. This number need not be exact: if we over-approximate the number of iterations, the resulting reach tube will further over-approximate the desired one.
6. We over-approximate the dynamics subject to general inputs (for parametric inputs or in the absence of inputs this step will be ignored), using the techniques described in Sect. 6.2.
7. We calculate the accelerated dynamics using the techniques described in Sect. 5.1.
8. We transform the input and initial sets into vertex representation, to be used as the source for the reach tube calculation.
9. We employ a sound simplex algorithm [16] to evaluate the convex-set Hadamard product of the abstract dynamics and of the initial set. The two most important elements of the sound simplex are that it uses interval arithmetic to pivot, and that at every stage it verifies the intersection between vertices in order to avoid pivoting on unsound solutions. The latter step is better explained by considering that the simplex algorithm operates by visiting adjacent vertices. The interval arithmetic ensure that the solution at the last visited vertex is sound, but if there is an intersection, the new pivot may choose to start on the intersecting vertex instead (which is not sound), thus, by checking the intersection and extending the interval to encompass both vertices, we retain soundness (see [16] for details).
10. Since we have operated in the eigenspace so far, we transform the reach tube back into the state space via multiplication by $\mathbb {S}$.

**Fig. 3:**
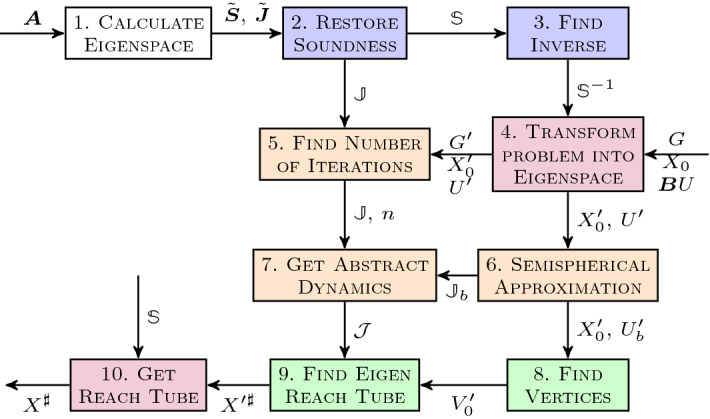
A block diagram describing the different steps used to calculate the abstract reach tube of a model via Abstract Acceleration. The white box is the numerical eigensolver stage. Blue boxes are soundness restoration stages. Red boxes represent linear transformations of the problem. The orange boxes denote abstractions defined in this paper, and the green boxes the reachability computation in the abstract domain. (Color figure online)

## Abstract Matrices in Abstract Acceleration

We introduce the concept of an abstract matrix.

### Definition 2

An abstract matrix ${\mathcal {A}}^n \subseteq \mathbb {R}^{p \times p}$ is an over-approximation of the union of the powers of the matrix $\varvec{A}^k$ such that ${\mathcal {A}}^n \supseteq \left\{ \varvec{A}^k : k\in [0, \ldots ,n] \right\}$. Its application to the initial set $X_0$ results in

9 $$\begin{aligned} {\hat{X}}_n^\sharp ={\mathcal {A}}^nX_0, \end{aligned}$$

such that ${\hat{X}}_n^\sharp \supseteq {\hat{X}}_n$ is an over-approximation of the reach tube described in Eq. (4).

Next we explain how to compute such abstract matrices. For simplicity, we first describe this computation for matrices $\varvec{A}$ with real eigenvalues, whereas the extension to the complex case will be addressed in Sect. 4.1. Similar to [44], we first have to compute the Jordan normal form of $\varvec{A}$. Let $\varvec{A}=\varvec{S}\varvec{J}\varvec{S}^{-1}$ where $\varvec{J}$ is the normal Jordan form of $\varvec{A}$, and $\varvec{S}$ is made up by the corresponding generalised eigenvectors. We can then easily compute $\varvec{A}^n=\varvec{S}\varvec{J}^n\varvec{S}^{-1}$, where given a set of *r* eigenvalues $\lambda _s$ with geometric multiplicities $p_s$ and $s \in [1,\ldots ,r]$, we have

10 $$\begin{aligned} \varvec{J}^n=&\left[ \begin{array}{ccc} \varvec{J}_1^n &{} &{} \\ &{} \ddots &{} \\ &{} &{} \varvec{J}_r^n\\ \end{array} \right] , \quad \text { where } \quad \varvec{J}_{s}^n= \left[ \begin{array}{cccc} \lambda _s^n &{} \left( {\begin{array}{c}n\\ 1\end{array}}\right) \lambda _s^{n-1} &{} \ldots &{} \left( {\begin{array}{c}n\\ p_s-1\end{array}}\right) \lambda _s^{n-p_s+1} \\ &{} \lambda _s^n &{} \left( {\begin{array}{c}n\\ 1\end{array}}\right) \lambda _s^{n-1} &{} \vdots \\ \vdots &{} &{} \ddots &{} \vdots \\ &{} &{} &{}\lambda _s^n \\ \end{array} \right] . \end{aligned}$$

The abstract matrix ${\mathcal {A}}^n$ is computed as an abstraction over a set of vectors $\varvec{m}^k \in \mathbb {R}^p, k \in [1,\ldots ,n]$ of distinct entries of $\varvec{J}^k$, as explained below.

Let $$\varvec{I}_s=[\begin{array}{cccc}1&0&\cdots&0\end{array}] \in \mathbb {R}^{p_s}$$. The vector $\varvec{m}^k$ is obtained by the transformation $\varphi ^{-1}$ (which is always invertible) as

11 $$\begin{aligned} \varvec{m}^k=\varphi ^{-1}\left( \varvec{J}^k\right) =\left[ \begin{array}{ccc}\varvec{I}_1 \varvec{J}_1^k&\cdots&\varvec{I}_r \varvec{J}_r^k\end{array}\right] ^\intercal \in \mathbb {R}^p, \end{aligned}$$

such that $$\varvec{J}^k = \varphi (\varvec{m}^k)$$.

If $\varvec{J}$ is diagonal [44], then $\varvec{m}^k$ results in the vector made up of powers of the eigenvalues, $$[\begin{array}{ccc}\lambda _1^k&\cdots&\lambda _p^k\end{array}]$$. The diagonal entries in the abstract matrix is thus bound by the intervals

12 $$\begin{aligned} \left\{ \begin{array}{ll} \left[ \min \{|\lambda _s|^0, |\lambda _s|^n\}, \max \{|\lambda _s|^0, |\lambda _s|^n\}\right] , &{} \lambda _s \in \mathbb {R}^+ \\ \left[ -\max \{|\lambda _s|^0, |\lambda _s|^n\}, \max \{|\lambda _s|^0, |\lambda _s|^n\}\right] , &{} \text { otherwise } \end{array}\right. , \text { where } s\in [1,\ldots ,r], r = p. \end{aligned}$$

We observe that the spectrum of the abstract matrix $\sigma ({\mathcal {A}}^n)$, which can be derived from its entries in Eq. (12), over-approximates $\bigcup _{k\in [1,\ldots ,n]} \sigma (\varvec{A}^k)$.

In the case of the $s\mathrm {th}$ Jordan block $\varvec{J}_s$ with geometric multiplicity $p_s > 1$, observe that the first row of $\varvec{J}_s^n$ contains all (possibly) distinct entries of $\varvec{J}_s^n$. Hence, the vector section $\varvec{m}_s$ is the concatenation of the (transposed) first row vectors $$\left( \lambda _s^n , \left( {\begin{array}{c}n\\ 1\end{array}}\right) \lambda _s^{n-1}, \ldots , \left( {\begin{array}{c}n\\ p_s-1\end{array}}\right) \lambda _s^{n-p_s+1}\right) ^\intercal $$ of $\varvec{J}_s^n$.

Since $\varphi$ transforms the vector $\varvec{m}$ into the shape of (10) of $\varvec{J}^n$, it is called a *matrix shape* [44]. We then define the abstract matrix as

13 $$\begin{aligned} {\mathcal {A}}^n = \{\varvec{S}\ \varphi (\varvec{m})\ \varvec{S}^{-1} : \varvec{\varPhi }\varvec{m} \le \varvec{f} \} \;, \end{aligned}$$

where the constraint $\varvec{\varPhi }\varvec{m} \le \varvec{f}$ is synthesised from intervals associated to the individual eigenvalues and to their combinations. More precisely, we compute polyhedral relations: for any pair of eigenvalues (or distinct entries) within $\varvec{J}$, we find an over-approximation of the convex hull containing the points

$$\begin{aligned} \left\{ \varvec{m}^k : k \in [1,\ldots ,n] \right\} \subseteq \left\{ \varvec{m} : \varvec{\varPhi }\varvec{m} \,{\le }\, \varvec{f} \right\} . \end{aligned}$$

The reason for evaluating the convex hull over pairs of points is twofold. In the first instance, we note that the set $\left\{ \varvec{m}^k \mid k \in [1,\ldots ,n] \right\}$ is, in general, not convex. This makes it hard to find its support in arbitrary directions. Ideal directions would be the normals to the gradients of the function, namely $\nabla m^k$, which would provide the tightest over-approximation at iteration *k*. However, as will be seen below, when combining negative or complex conjugate eigenvalues, the corresponding hyperplane tangent may intersect the set, and thus it cannot be used to define its convex hull. The second reason for choosing pairwise directions is practical: we need an even distribution of the directions in $\mathbb {R}^p$, and it is easier to do this in a pairwise manner.^1^

### Abstract Matrices in Complex Spaces

To deal with complex numbers in eigenvalues and eigenvectors, [44] employs the real Jordan form for conjugate eigenvalues $\lambda = re^{i\theta }$ and $\lambda ^* = re^{-i\theta }$ ($\theta \in [0, \pi ]$), so that

$$\begin{aligned} \left[ \begin{array}{@{}cc@{}}{\lambda }&{}{0}\\ {0}&{}{ \lambda ^*}\end{array}\right] \quad \text { is replaced by } \quad r \left[ \begin{array}{@{}cc@{}}{\cos \theta }&{}{\sin \theta }\\ {-\sin \theta }&{}{\cos \theta }\end{array}\right] . \end{aligned}$$

Although this equivalence will be of use once we evaluate the progression of the model, calculating powers under this notation is often more difficult than handling directly the original matrices with complex values.

In the case of real eigenvalues we have abstracted the entries in the power matrix $\varvec{J}_s^n$ by ranges of eigenvalues $[\min \{\lambda _s^0 \cdots \lambda _s^n\}, \max \{\lambda _s^0 \cdots \lambda _s^n\} ]$, forming a hypercube. In the complex case, where the rotations describe spherical behaviour, we can do something similar by rewriting eigenvalues into the polar form $\lambda _s = r _s e^{i\theta _s}$ and enclosing the radius in the interval $[0, {\overline{r}}_s]$, where ${\overline{r}}_s=\max \{r_s^k : k \in [0,\ldots ,n]\}$ (in the worst case scenario this is over-approximated by a hyper-box with $\lambda _s^k \in [-{\overline{r}}_s, {\overline{r}}_s]+[-{\overline{r}}_s, {\overline{r}}_s]i$, but we will introduce tighter bounds in the course of this work).

## Abstract Acceleration Without Inputs

### Using Support Functions for Abstract Acceleration

As an improvement over [44], the rows in $\varvec{\varPhi }$ and $\varvec{f}$ (see (13)) can be obtained by a refined sampling of the support functions of these sets. The choice of directions for these support functions results in an improvement over the logahedral abstractions used in previous work [37, 38, 44] (see Figs. 4,5, 6 and 7). This approach works thanks to the convex properties of the exponential progression. We consider five cases:

1. *Positive Real Eigenvalues* The exponential curve is cut along the diagonal between the eigenvalues with maximum and minimum range to create a supporting hyperplane. A third point taken from the curve is used to test the direction of the corresponding template vector. An arbitrary number of additional supporting hyperplanes are created by selecting pairs of adjacent points in the curve and creating the corresponding support functions, as illustrated in Fig. 4.
2. *Complex Conjugate Eigenvalue Pairs* In the case of complex conjugate pairs, the eigenvalue map corresponds to a logarithmic spiral (Fig. 5). In this case, we must first extract the number of iterations (denoted by ${\overline{k}}$) required for a full cycle. For convergent eigenvalues ($|{\lambda }|<1$), only the first ${\overline{k}}$ iterations have an effect on the support functions, while in the divergent case only the last ${\overline{k}}$ iterations are considered (since symmetrically, it corresponds to the reverse spiral case). Support functions are found for adjacent pairs, checking the location of the first point for convergent eigenvalues, and that of the last point for divergent eigenvalues. If a point falls outside of the supporting half-space, we look for an interpolant point that closes the spiral and that is tangent to the origin. This last check is performed as a binary search over the remaining points in the circle (noting that the supporting planes would exclude the considered point) to achieve maximum tightness (Fig. 5).
  Care is taken to ensure that subsequent iterations fall within the envelope found on the first/last rotation. This is ensured by extending the support functions outwards by a factor $f = \max \left( \{1,|\lambda |^{{\hat{n}}}\cos (\theta )^{-1}\}\right)$, where $\theta$ is the angle of the eigenvalue pair and ${\hat{n}}=n$ for the convergent case or ${\hat{n}}=\frac{1}{n}$ for the divergent case. When this value is too large, we use an interpolation to find better supports. This is achieved by finding a pair such that the first point is obtained from $\lambda ^k$ and the second from $(\lambda ^{\frac{1}{m}})^{mk+1}$. The relaxation factor then becomes $\cos \left( \frac{\theta }{m}\right) ^{-1}$.
3. *Equal Eigenvalues* When two eigenvalues are equivalent, the resulting support functions are those that are orthogonal to the $x=y$ plane, intersecting the square created by the maximum and minimum values.^2^
4. *Jordan Blocks of Non-trivial Size* ($>1$) In the case of eigenvalues with geometric multiplicities, we find three shapes. When both elements in the pair are convergent (convex sets can be “sharp”), it is important to find the apex of the upper diagonals in order to minimise the over-approximation (Fig. 6). When both elements are divergent, the shape is similar to a positive valued pair since there is no extremum. Finally, when comparing different Jordan blocks, one convergent and one divergent, we evaluate the enclosing hyperbox, thus avoiding the change in convexity at the apex.
5. *Negative Eigenvalues and Combinations of Real Eigenvalues with Conjugate Pairs* When comparing a positive real eigenvalue to a complex conjugate or a negative one, we must account for the changes of sign in the progression of the latter. We compute envelopes of the progression of the corresponding dynamics, which are obtained via convex over-approximations (cf. Fig. 7). In the case of complex eigenvalues, we use the absolute value in order to determine the envelope. If both eigenvalues have rotating dynamics, we would require full symmetry along the four quadrants, and thus we obtain a hyper-box with vertices at the farthest points from the origin.

**Fig. 4:**
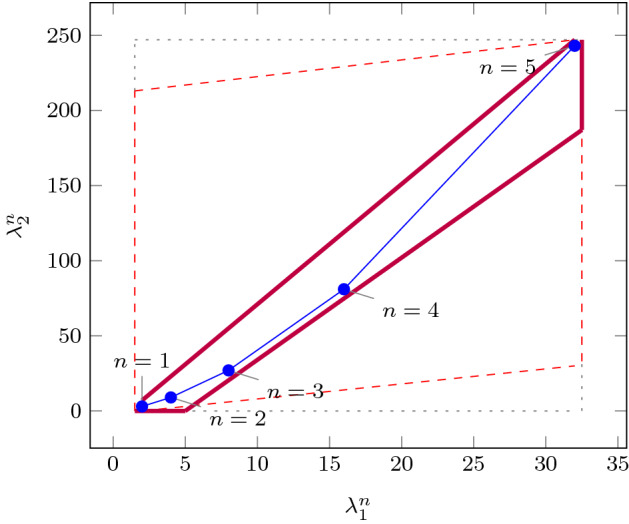
Polyhedral faces over $\mathbb {R}^2$ for pairs of eigenvalues $(\lambda _1^n, \lambda _2^n)$ where $\lambda _1=2, \lambda _2 =3$, and $1{\le }n{\le }5$. Bold purple lines represent supports. The dotted grey and dashed red polytopes show logahedral approximations (box and octagon) used in [44]. Note the scales (the sloped dashed lines are parallel to the x = y line, and the dashed red polytope hides two small faces yielding an octagon). (Color figure online)

**Fig. 5:**
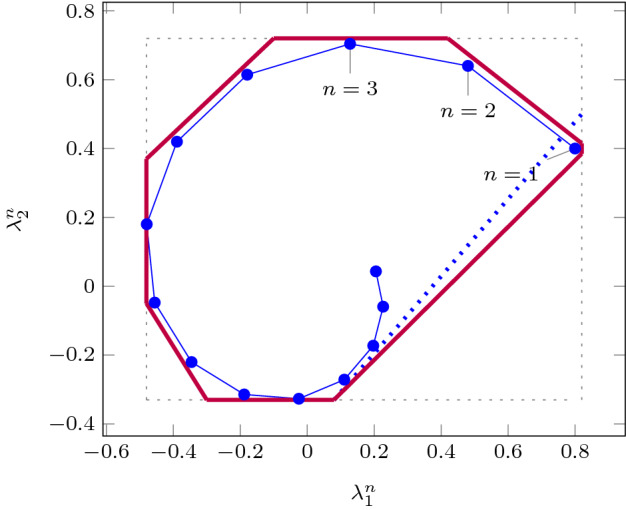
Polyhedral faces projected onto $\mathbb {R}^2$ for complex conjugate eigenvalues $(\lambda _1^n, \lambda _2^n)$ where $\lambda _1=0.8+0.4i, \lambda _2=0.8-0.4i$, and $1{\le }n{\le }14$. Bold purple lines represent supports. The blue dotted line shows the supporting hyperplane that excludes the point obtained with $n=1$, which is replaced by a supporting hyperplane tangent to the spiral but touching said origin. (Color figure online)

**Fig. 6:**
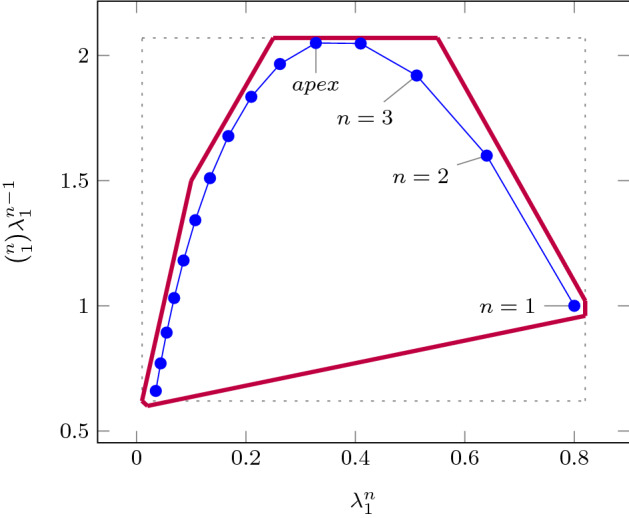
Polyhedral faces in $\mathbb {R}^2$ related to a Jordan block $$(\lambda _1^n, \left( {\begin{array}{c}n\\ 1\end{array}}\right) \lambda _1^{n-1})$$, where $\lambda _1=0.8$ and $1{\le }n{\le }15$. Bold purple lines represent supports found in this work. (Color figure online)

**Fig. 7:**
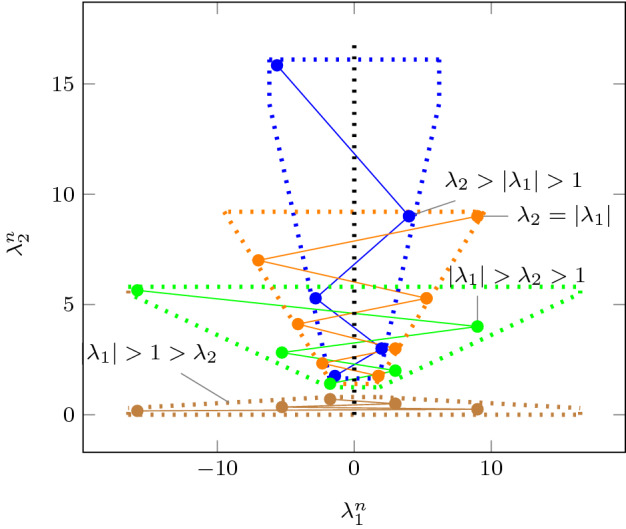
Polyhedral faces over $\mathbb {R}^2$, of different eigenvalue ratios (note that the curves obtained from the progression of the blue and orange dots are convex w.r.t. the $\lambda _2^n$-axis, whereas the green and brown are concave). Dotted lines represent convex supports for these layouts. (Color figure online)

An additional drawback of [44] is that calculating the exact Jordan form of any matrix is computationally expensive for high-dimensional matrices. We will instead leverage numerical algorithms that provide an approximation of the Jordan normal form and soundly account for the associated numerical errors. We use properties of eigenvalues to relax $\varvec{f}$ by finding the maximum error in the calculations, which can be determined by computing the norm $\delta _{max} = \Vert \hat{\mathbb {S}}\hat{\mathbb {J}}\hat{\mathbb {S}}^{-1}-\mathbb {A}\Vert$, where $\hat{\mathbb {J}}$ and $\hat{\mathbb {S}}$ are the eigenvalues and eigenvectors of $\varvec{A}$ calculated numerically [16]. Recall that the notation above is used to represent interval matrices, and that all operations are performed using interval arithmetic with outward rounding in order to ensure soundness. The constraints in $\varvec{\varPhi } \varvec{m} \le \varvec{f}$ are then computed by considering the ranges of eigenvalues $|\lambda _s \pm \delta _{max}|^k$, which are represented in Fig. 4 with blue circles.

The outward relaxation of the support functions ($\varvec{f}$) follows a principle similar to that in [33], and reduces the tightness of the over-approximation, while ensuring the soundness of the obtained abstract matrix ${\mathcal {A}}^n$. Graphically, this is equivalent to moving the faces of a polyhedron outward, which practically has a minimal impact due to the small magnitude of $\delta _{max}$. It is also worth noting that the transformation matrices into and from the eigenspace will also introduce over-approximations due to the intervals, and will exacerbate the over-approximations due to the condition number related to the eigenvalues.

One can still use exact arithmetic with a noticeable improvement over previous work; however, for higher-dimensional models the option of using floating-point arithmetic (taking errors into account and meticulously setting rounding modes) provides a 100-fold improvement, which can render verification practically feasible. For a full description of the numerical techniques see [16].

### Abstract Matrices and Support Functions

Since we are describing operations using abstract matrices and support functions, we briefly review the nature of these operations and the properties that support functions retain within this domain. Let $X \in \mathbb {R}^p$ be a set and ${\mathcal {A}} \in {\mathcal {R}}^{p \times p}$ an abstract matrix for the same space. From Eq. (13) we have

$$\begin{aligned} {\mathcal {A}}=\bigcup \varvec{S}\varphi (\varvec{m})\varvec{S}^{-1} \text {, where } \varvec{\varPhi }\varvec{m} \le \varvec{f}, \end{aligned}$$

which leads to

14 $$\begin{aligned} \rho _{{\mathcal {A}}X}(\varvec{v})=\rho _{\varvec{S}\varphi (\varvec{m})\varvec{S}^{-1}X}(\varvec{v})=\rho _{\varphi (\varvec{m})\varvec{S}^{-1}X}\left( \varvec{S}^\intercal \varvec{v}\right) , \end{aligned}$$

where $\rho _{\varphi X}(\varvec{v}) = \sup \left\{ \rho _{\varphi }(\varvec{x} \circ \varvec{v}) : \varvec{x} \in X\right\} ,$ and $\rho _{\varphi }(\varvec{v}) = \sup \{\varvec{m} \cdot \varphi ^{-1}(\varvec{v}) : \varvec{\varPhi }\varvec{m} \le \varvec{f}\}$. Here, $\varvec{x} \circ \varvec{y}$ is the Hadamard product, where $(\varvec{x} \circ \varvec{y})_{i}=\varvec{x}_i\varvec{y}_i$, and $\varphi ^{-1}(\cdot )$ is the inverse operation of $\varphi (\cdot )$. We also define

$$\begin{aligned} \rho _{{\mathcal {A}}X}(\varvec{v})&=\sup \left\{ \rho _{\varvec{a}X}(\varvec{v}),\ \varvec{a} \in {\mathcal {A}}\right\} \nonumber \\&=\sup \left\{ \varvec{S}\varphi (\varvec{m})\varvec{S}^{-1}\varvec{x} \cdot \varvec{v},\ \varvec{x} \in X, \varvec{\varPhi }\varvec{m} \le \varvec{f}\right\} \nonumber \\&=\sup \left\{ \varphi (\varvec{m})\varvec{S}^{-1}\varvec{x} \cdot \varvec{S}^\intercal \varvec{v},\ \varvec{x} \in X, \varvec{\varPhi }\varvec{m} \le \varvec{f}\right\} \nonumber \\&=\sup \left\{ \rho _{\varphi }(\varvec{S}^{-1}\varvec{x} \circ \varvec{S}^\intercal \varvec{v}),\ \varvec{x} \in X\right\} , \end{aligned}$$

and, in order to simplify the nomenclature, we write

15 $$\begin{aligned} \rho _{{\mathcal {A}}X}(\varvec{v})=\rho _{X}({\mathcal {A}}^\intercal \varvec{v}). \end{aligned}$$

## General Abstract Acceleration with Inputs

### Acceleration of Parametric Inputs

Let us now consider the following over-approximation for $\tau$ on sets:

16 $$\begin{aligned} \tau ^\sharp (X_0,U) = \varvec{A}X_0\oplus \varvec{B}U, \end{aligned}$$

and add a restriction to constant (also called parametric) inputs, namely $\varvec{u}_k = \varvec{u}_0, \forall k>0$ and $\varvec{u}_0 \in U$. Unfolding (3) (ignoring the presence of the guard set *G* for the time being), we obtain

17 $$\begin{aligned} X_n=\varvec{A}^nX_0\oplus \sum _{k=0}^{n-1} \varvec{A}^k\varvec{B}U. \; \end{aligned}$$

We further simplify the sum $\sum _{k=0}^{n-1} \varvec{A}^k\varvec{B}U$, exploiting the following result from linear algebra.

#### Lemma 2

If $\varvec{I}-\varvec{A}$ is invertible, then

$$\begin{aligned} \sum \limits _{k=0}^{n-1} \varvec{A}^k = (\varvec{I} -\varvec{A}^{n})(\varvec{I} - \varvec{A})^{-1}. \end{aligned}$$

If furthermore $\lim \limits _{n\rightarrow \infty } \varvec{A}^n=0$, then $\lim \limits _{n \rightarrow \infty } \sum \limits _{k=0}^n \varvec{A}^k = (\varvec{I}-\varvec{A})^{-1}.$

The inverse $(\varvec{I}-\varvec{A})^{-1}$ does not exist for eigenvalues equal to 1, i.e., we need $1\notin \sigma (\varvec{A})$, where $\sigma (A)$ is the spectrum (the set of all the eigenvalues) of matrix $\varvec{A}$. In order to address this problem, we introduce the eigendecomposition of $\varvec{A} = \varvec{S}\varvec{J}\varvec{S}^{-1}$, (and trivially $\varvec{I}=\varvec{S}\varvec{I}\varvec{S}^{-1}$), and by the distributive and transitive properties we obtain

$$\begin{aligned} (\varvec{I} -\varvec{A}^{n})(\varvec{I} - \varvec{A})^{-1} = \varvec{S}(\varvec{I}-\varvec{J}^{n})(\varvec{I}-\varvec{J})^{-1}\varvec{S}^{-1} \;. \end{aligned}$$

Although $(\varvec{I}-\varvec{J})$ is still not invertible, this representation allows us to accelerate the eigenvalues individually, trivially noticing that $\sum _{k=0}^{n-1} 1^k = n$ for unitary eigenvalues (thus eliminating the need to calculate said inverse for these eigenvalues). Using the properties above, and translating the problem into the generalised eigenspace to account for unit eigenvalues, we obtain the following representation:

18 $$\begin{aligned} (\varvec{I}-\varvec{A}^n)(\varvec{I}-\varvec{A})^{-1}&= \varvec{S}\varvec{D}^n\varvec{S}^{-1}, \end{aligned}$$

given

$$\begin{aligned} \displaystyle \varvec{D}^n_{i,j}&=\left\{ \begin{array}{ll} 0 &{}\quad gm(\lambda _i) \le k \vee n<k\\ d(\lambda _i,n,0) &{}\quad i=j\\ \left( {\begin{array}{c}n+1\\ k+1\end{array}}\right) &{} \lambda _i = 1 \\ d(\lambda _i,n,j-i) &{} \lambda _i \ne 1, \\ \end{array} \right. \end{aligned}$$

where

$$\begin{aligned} d(\lambda _i,n,0)&= \sum _{k=0}^{n-1} \lambda _i^k = \left\{ \begin{array}{ll} n &{}\quad \lambda _i =1 \\ \frac{1-\lambda _i^n}{1-\lambda _i} &{} \quad \lambda _i \ne 1,\\ \end{array} \right. \nonumber \\ d(\lambda _i,n,k)&=\frac{-1^k}{k+1}\frac{1-\lambda _i^n}{(1-\lambda _i)^{k+1}} +\sum \limits _{j=1}^{k}\frac{-1^{k-j}}{k-j}\left( {\begin{array}{c}n\\ j-1\end{array}}\right) \frac{\lambda _i^{n-j-1}}{(1-\lambda _i)^{k-j}}, \end{aligned}$$

and where $gm(\cdot )$ denotes the geometric multiplicity of the given eigenvalue, and $k=j-i$. With these notions in hand, we next define the abstract acceleration of parametric inputs.

#### Theorem 2

The abstract acceleration is defined as

19 $$\begin{aligned} {\hat{\tau }}^{\sharp n}(X_0,U) =_{\mathrm {def}} {\mathcal {A}}^nX_0 \oplus {\mathcal {B}}^n U, \end{aligned}$$

where ${\mathcal {B}}^n\supseteq \bigcup _{k \in [1,\ldots ,n]}\varvec{S}(\varvec{D}^k)\varvec{S}^{-1}\varvec{B}$, is an over-approximation of the *n*-reach tube, namely ${\hat{X}}_n \subseteq {\hat{\tau }}^{\sharp n}(X_0,U)$.

#### Proof

From Eq. (4) we have

$$\begin{aligned} {\hat{X}}_n = {\hat{\tau }}^n(X_0,U) =\bigcup _{k\in [0,\ldots ,n]} \tau ^k(X_0,U). \end{aligned}$$

Using Eq. (17), we expand this into

$$\begin{aligned} {\hat{X}}_n=\bigcup _{k\in [0,\ldots ,n]}\varvec{A}^kX_0\oplus \sum _{j=0}^{k-1} \varvec{A}^j\varvec{B}U \subseteq \bigcup _{k\in [0,\ldots ,n]}\varvec{A}^kX_0\oplus \bigcup _{k\in [0,\ldots ,n]}\sum _{j=0}^{k-1} \varvec{A}^j\varvec{B}U, \end{aligned}$$

then replace

$$\begin{aligned} {\hat{X}}_n \subseteq {\mathcal {A}}^nX_0 \oplus \bigcup _{k \in [1,\ldots ,n]}\varvec{S}(\varvec{D}^k)\varvec{S}^{-1}\varvec{B}U \end{aligned}$$

and finally obtain

$$\begin{aligned} {\hat{X}}_n \subseteq {\hat{\tau }}^{\sharp n}(X_0,U). \end{aligned}$$

The quantities ${\mathcal {A}}^n$ and ${\mathcal {B}}^n$ are calculated using the techniques described in Sect. 5.1, where special consideration is taken to evaluate pairs comprising equal eigenvalues. Figure 8 gives an example of such a pair. Since both functions are monotonic, the set is convex. The techniques applied to positive real eigenvalues (see Sect. 5.1) therefore stands. $\square$

**Fig. 8:**
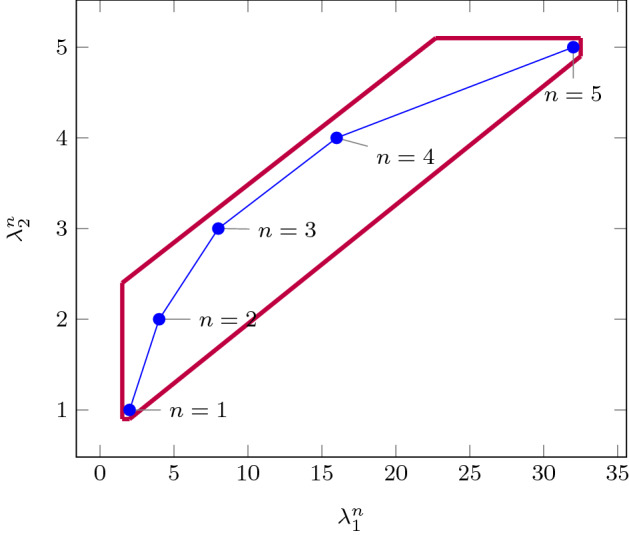
Polyhedral faces $(\lambda _1^n, n)$ over $\mathbb {R}^2$, where $\lambda _1=2, \lambda _2=3$, and $1{\le }n{\le }5$. Bold purple lines represent supports found in this work. (Color figure online)

### Acceleration of Time-Varying Inputs

In order to generalise the previous results for use with time-varying inputs, we will over-approximate the term $\varvec{B}U$ over the eigenspace by a semi-spherical enclosure, namely a set where complex conjugate eigenvalues are enclosed by a spherical support with centre $\varvec{u}_c'$ and the radius of $U_b'$, whereas real eigenvalues are enclosed by hyper-rectangles (dashed symbols represent elements in the eigenspace). To this end, we first rewrite

$$\begin{aligned} U_J' = \varvec{S}^{-1}\varvec{B}U = \{ \varvec{u}_c'\} \oplus U_d', \end{aligned}$$

where $\varvec{u}_c'$ is the centre of the smallest hyperbox (interval hull) containing $U_J'$, and $U_d'=\{ \varvec{u} : \varvec{u}+\varvec{u}_c' \in U_J'\}$ as:

20 $$\begin{aligned} {\varvec{u}_c'}_i = \frac{1}{2}(\rho _{U_J'}(\varvec{v_i})+\rho _{U_J'}(-\varvec{v_i})), \text { where } {\varvec{v}_i}_j= \left\{ \begin{array}{ll} 1 &{}\quad j=i\\ 0 &{}\quad j \ne i \end{array} .\right. \end{aligned}$$

We then over-approximate $U_d'$ via $U_b'$, by the maximum radius in the directions of the complex eigenvalues (cf. illustration in Fig. 9). Let

$$\begin{aligned} \varLambda&=\{\lambda _i: im(\lambda _i) \ge 0\} \end{aligned}$$

be the set of eigenvalues of $\varvec{A}$, where $im(\cdot )$ is the imaginary value of a complex number, and conjugate pairs are represented only by one member of the pair. Let us define the function $f_b:\mathbb {R}^p \rightarrow \mathbb {R}^{p_b}$, where $p_b$ is the cardinality of $\varLambda$, such that

$$\begin{aligned} f_b(\varvec{v})&=\text {red}(\varvec{v}_b), \text { where } (\varvec{v}_b)_i=\left\{ \begin{array}{ll} 0 &{}\quad \lambda _i \notin \varLambda \\ |\varvec{v}_i| &{}\quad \lambda _i \ne \lambda _{i+1}^*\\ \sqrt{\varvec{v}_i^2+\varvec{v}_{i+1}^2} &{}\quad \lambda _i = \lambda _{i+1}^* \end{array} \right. , \end{aligned}$$

$i \in [1,\ldots ,p]$ and red($\cdot$) is a function that reduces the dimension of a vector by removing the elements where $\lambda _i \notin \varLambda$ (*i.e.* the null elements in $\varvec{v}_b$, such that if for instance $$\varvec{v}_b = [\begin{array}{ccccc}v_1&0&v_3&\ldots&v_p\end{array}]^\intercal $$, then $$\text {red} (\varvec{v}_b) = [\begin{array}{cccc}v_1&v_3&\ldots&v_p\end{array}]^\intercal $$). Extending this to matrices we have

$$\begin{aligned} F_b : \mathbb {R}^{r \times p} \rightarrow \mathbb {R}^{r \times p_b}, \quad F_b(\varvec{C}) = \varvec{C}_b \text { where } ({\varvec{C}_b})_{i,*}=f_b(\varvec{C}_{i,*}), \end{aligned}$$

where *r* denotes the number of inequalities describing a set in $\mathbb {R}^p$. Finally,

21 $$\begin{aligned} U_d'&=\{ \varvec{u} \mid \varvec{C}_u'\varvec{u} \le \varvec{d}_u' \}, U_d' \subseteq U_b', \text { with } \nonumber \\ U_b'&= \{\varvec{u} \mid F_b(\varvec{C}_u')f_b(\varvec{u}) \le f_b(\varvec{d}_u') \}, \text { and }\nonumber \\ \varvec{B}U&\subseteq U_b \oplus U_c, \text { where } U_b=\varvec{S}U_b' \text { and } U_c=\{\varvec{S}\varvec{u}_c'\}. \end{aligned}$$

Since the description of $U'_b$ is no longer polyhedral in $\mathbb {R}^p$, we will also create an over-approximation $\varvec{J}_b$ of $\varvec{J}$ in the directions of the complex eigenvectors, in way that is similar to how we generated $U_b'$ for $U_d'$. More precisely,

22 $$\begin{aligned} \varvec{J}_b&=\left[ \begin{array}{ccc} \varvec{J}_{b1} &{} &{} \\ &{} \ddots &{} \\ &{} &{} \varvec{J}_{br}^n\\ \end{array} \right] ,\;\; \text { where } \forall s \in [1,\ldots ,r]\; \left\{ \begin{array}{l}\lambda _j \in {\varvec{J}_b}_s = |\lambda _i| \in \varvec{J}_s \cap \varLambda \\ gm({\varvec{J}_b}_s)=gm(\varvec{J}_s),\end{array}\right. \end{aligned}$$

and $gm(\cdot )$ is the geometric multiplicity of the specific Jordan block.

#### Definition 3

Given a matrix $\varvec{A}=\varvec{S}\varvec{J}\varvec{S}^{-1}$ and a vector $\varvec{x}$, we define the following operations:

23 $$\begin{aligned} F_b^*(\varvec{A},\varvec{x})&=\varvec{S}f_b^{-1}\left( F_b(\varvec{J})f_b(\varvec{S}^{-1}\varvec{x})\right) . \end{aligned}$$

Finally, we refer to the accelerated sets

$$\begin{aligned} U_b^n&=\left\{ F_b^*((\varvec{I}-\varvec{A}^n),F_b^*((\varvec{I}-\varvec{A})^{-1},\varvec{u})) \mid \varvec{u} \in U_b \right\} , \\ U_c^n&=(\varvec{I}-\varvec{A}^n)(\varvec{I}-\varvec{A})^{-1}U_c,\quad U_{cb}^n =U_c^n \oplus U_b^n. \end{aligned}$$

Returning to our original equation for the *n*-reach set, we obtain^3^

24 $$\begin{aligned} X_n \subseteq \varvec{A}^nX_0&\oplus U_{cb}^n. \end{aligned}$$

**Fig. 9:**
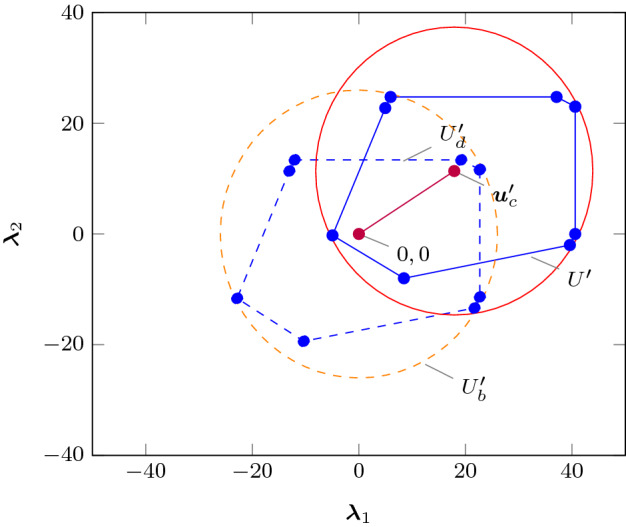
Relaxation of an input set within a complex subspace, in order to make it invariant to matrix rotations. Dashed lines and curves denote translated quantities onto the origin

Shifting our attention from reach sets to reach tubes, we can now over-approximate the reach tube by abstract acceleration of the summands in (24), as follows.

#### Theorem 3

The abstract acceleration

25 $$\begin{aligned} {\hat{\tau }}^{\sharp n}(X_0,U) = {\mathcal {A}}^nX_0 \oplus {\mathcal {B}}^n U_c \oplus {\mathcal {B}}_b^n U_b, \end{aligned}$$

where ${\mathcal {A}}^n\supseteq \bigcup _{k \in [1,\ldots ,n]}\varvec{A}^k$, ${\mathcal {B}}^n\supseteq \bigcup _{k \in [1,\ldots ,n]}\sum _{i=0}^{k-1}\varvec{A}^i\varvec{B}$, and ${\mathcal {B}}_b^n\supseteq \bigcup _{k \in [1,\ldots ,n]}F_b^*\left( \sum _{i=0}^{k-1}\varvec{A}^i\varvec{B},\varvec{x}\right)$, denotes an over-approximation of the *n*-reach tube, namely ${\hat{X}}_n \subseteq {\hat{\tau }}^{\sharp n}(X_0,U)$.

#### Proof

From Eq. (24) we have that $X_n \subseteq \varvec{A}^nX_0 \oplus U_{cb}^n = \varvec{A}^nX_0 \oplus U_c^n \oplus U_b^n$. Furthermore, from Eq. (24) we also have ${\hat{\tau }}^{\sharp n}(X_0,U_c) \supseteq \bigcup _{k \in [1,\ldots ,n]} \varvec{A}^kX_0 \oplus U_c^k$. Finally, from the definition of ${\mathcal {B}}_b^n$ we have ${\mathcal {B}}_b^nU_b \supseteq \bigcup _{k \in [1,\ldots ,n]}U_b^k$, hence ${\hat{\tau }}^{\sharp n}(X_0,U) \supseteq {\hat{X}}_n$. $\square$

### Combining Abstract Matrices

Calculating the reach set from the set of initial states and that originating from the input set separately, and then adding them together, can result in coarse over-approximations. To minimise this effect, we apply abstract acceleration to the combined input-and-state spaces.

One important property of the abstract matrices ${\mathcal {A}}^n$, ${\mathcal {B}}^n$ and ${\mathcal {B}}_b^n$ is that they are related. In the case of parametric inputs, this correlation is linear and is covered by the acceleration defined in Lemma (). In the case of ${\mathcal {B}}_b^n$ this relationship is not linear (see Eq. 21). However, we can still find a linear over-approximation of the relation between ${\mathcal {B}}_b^n$ and ${\mathcal {A}}^n$ based on the time steps *k*.

Given two sets $X \in \mathbb {R}^p$ and $U \in \mathbb {R}^q$ and a transition equation $X_{k+1}=\varvec{A}X_k + \varvec{B}U$, which is related to $\rho _{X_{k+1}}(\varvec{v})=\rho _{\varvec{A}X_k}(\varvec{v}) + \rho _{\varvec{B}U}(\varvec{v})$, we define a set

$$\begin{aligned} X'=\left\{ \left[ \begin{array}{@{}c@{}}{\varvec{x}}\\ {\varvec{B}\varvec{u}}\end{array}\right] : \varvec{x} \in X, \varvec{u} \in U \right\} \end{aligned}$$

such that

$$\begin{aligned} \rho _{X_{k+1}}(\varvec{v})=\rho _{X'_k}\left[ \begin{array}{@{}c@{}}{\varvec{A}^\intercal \varvec{v}}\\ {\varvec{v}}\end{array}\right] =\rho _{X'_k}\left( \varvec{D}^\intercal \varvec{v}'\right) , \text { with } \varvec{D}=\left[ \begin{array}{@{}cc@{}}{\varvec{A}}&{}{0}\\ {0}&{}{\varvec{I}}\end{array}\right] , \quad \varvec{v}'= \left[ \begin{array}{@{}c@{}}{\varvec{v}}\\ {\varvec{v}}\end{array}\right] . \end{aligned}$$

Accelerating $X_{k+1}$, we obtain

$$\begin{aligned} \rho _{X_{n}}(\varvec{v})= & {} \rho _{\varvec{A}^nX_0}(\varvec{v}) + \rho _{(\varvec{I}-\varvec{A}^{n})(\varvec{I}-\varvec{A})^{-1}\varvec{B}U}(\varvec{v})=\rho _{X'_0}\left( (\varvec{D}^{n})^T\varvec{v}'\right) , \\&\quad \text { with } \varvec{D}^n=\left[ \begin{array}{@{}cc@{}}{\varvec{A}^n}&{}{0}\\ {0}&{}{(\varvec{I}-\varvec{A}^n)(\varvec{I}-\varvec{A})^{-1}}\end{array}\right] , \end{aligned}$$

in the case of parametric inputs. More generally, the diagonal elements of $\varvec{D}^n$ correspond to the diagonal elements of $\varvec{A}^n$ and $\sum _{k=0}^{n-1}{\varvec{A}^k} \varvec{B}$, which means we can construct

26 $$\begin{aligned} {\mathcal {D}}^n=\left[ \begin{array}{@{}cc@{}}{{\mathcal {A}}^n}&{}{0}\\ {0}&{}{{\mathcal {B}}^n}\end{array}\right] \text { such that } \rho _{X_n}(\varvec{v})=\rho _{X'_0}\left( ({\mathcal {D}}^n)^\intercal \varvec{v}'\right) , \end{aligned}$$

where ${\mathcal {A}}^n$ and ${\mathcal {B}}^n$ are the abstract matrices in Eqs. (13) and (19). We can then apply this abstraction to (21) and obtain:

27 $$\begin{aligned} \rho _{X_n}(\varvec{v})&=\rho _{X'_0}({\mathcal {D}}_b^{nT}\varvec{v}'), \text { where }\\ {\mathcal {D}}_b^n&=\left[ \begin{array}{@{}cc@{}}{{\mathcal {A}}^n}&{}{0}\\ {0}&{}{{\mathcal {B}}_b^n}\end{array}\right] \ ,\ \ \varvec{v}'= \left[ \begin{array}{@{}c@{}}{\varvec{v}}\\ {f_b(\varvec{v})}\end{array}\right] , \nonumber \\ {\mathcal {B}}_b^n&= \varvec{S}F_b^{-1}\left( (\varvec{I}-{\mathcal {J}}_b^n)(\varvec{I}-\varvec{J}_b)^{-1}F_b(\varvec{S}^{-1})\right) ,\nonumber \end{aligned}$$

with $\varvec{J}_b$ defined in (22). This model provides a tighter over-approximation than (25), since the accelerated dynamics of the inputs are now coupled to the acceleration of the dynamical part of the model.

#### Example 1

In order to illustrate this, let us consider the one-dimensional model $\varvec{x}_{k+1}=0.5\varvec{x}+1$ with initial state $\varvec{x}_0=1$. If we calculate ${\mathcal {A}}$ and ${\mathcal {B}}$ separately we get $\hat{\varvec{x}}=\bigcup _{k=0}^{\infty } \varvec{A}^{k}\varvec{x}_0+\bigcup _{k=0}^{\infty } (1-\varvec{A}^{k})\frac{\varvec{u}}{1-\varvec{A}} = [1, 3]$, however, using ${\mathcal {D}}$ we have $\hat{\varvec{x}}=\bigcup _{k=0}^{\infty } \varvec{A}^{k}\left( \varvec{x}_0-\frac{\varvec{u}}{1-\varvec{A}}\right) +\frac{\varvec{u}}{1-\varvec{A}} = [1, 2]$. $\square$

## Abstract Acceleration with Guards: Estimation of the Number of Iterations

In the presence of spatial guards *G*, we are interested in estimating the number of iterations used to calculate the abstract matrices. Since we are dealing with reach sets, we differentiate between sets that are entirely inside the guard, sets that are crossing it, and sets that are entirely outside. The latter reach sets should never be propagated, whereas reach sets crossing guards should be made as tight as possible.

Given a convex polyhedral guard expressed as the assertion $G=\{\varvec{x} : \varvec{G}\varvec{x} \le \varvec{h}\}$, we define $G_{i,*}$ as the $i{\text {th}}$ row of $\varvec{G}$ and $h_i$ as the corresponding element of $\varvec{h}$. We denote the normal vector to the $i{\text {th}}$ face of the guard as $\varvec{g}_i=G_i^\intercal$. The distance of the hyperplane defined by the i-th guard to the origin is thus $\gamma _i = \frac{h_i}{|\varvec{g}_i|}$.

Given a convex set *X*, we can now describe its position with respect to each face of the guard through the use of its support function alongside the normal vector to the hyperplane (for clarity, we assume that the origin is inside the set *X*):

$$\begin{aligned} \begin{array}{ccl} \rho _X(\varvec{g}_i) \le \gamma _i, &{} &{} \text {inside the hyperplane},\\ -\rho _X(-\varvec{g}_i) \ge \gamma _i, &{} &{} \text {outside the hyperplane}. \end{array} \end{aligned}$$

Applying this to Eq. (24) we obtain:

28 $$\begin{aligned}&\rho _{X_n}(\varvec{g}_i) =\rho _{X_0}({\varvec{A}^{\underline{n_i}}}^\intercal \varvec{g}_i)+\rho _{U_{cb}^n}(\varvec{g}_i) \le \gamma _i, \end{aligned}$$

29 $$\begin{aligned}&\rho _{X_n}(-\varvec{g}_i)=\rho _{X_0}(-{\varvec{A}^{\overline{n_i}}}^\intercal \varvec{g}_i)+\rho _{U_{cb}^n}(-\varvec{g}_i) \le -\gamma _i. \end{aligned}$$

From the inequalities above we can determine the number of iterations $\underline{n_i}$ for which the reach tube remains inside the corresponding hyperplane, and from which iteration $\overline{n_i}$ the corresponding reach set goes beyond the guard.

Therefore, in order for a reach set to be inside the guard it must be inside all of its faces, and we can ensure it is fully outside of the guard set when it is fully beyond any of them. Thus, we have ${\underline{n}} = \min \{\ \underline{ n_i }\ \}$ and ${\overline{n}} = \min \{\ \overline{ n_i }\ \}$.

We now discuss why these two cases are important. Looking at the transition in Eq. (1), we can easily derive that if $\varvec{G}\varvec{x}_k \not \le \varvec{h}$ (i.e., the point lies outside at least one of the faces of the guard set), the post-image of all subsequent iterations of that point must not be included. As such, any over-approximation of the reach set will only add imprecision. Therefore, we will use the bounds ${\underline{n}}$ and ${\overline{n}}$ to create a tighter over-approximation. Let

$$\begin{aligned} {\hat{X}}_{{\underline{n}}}^{\sharp }&={\mathcal {A}}^{{\underline{n}}}X_0\oplus {\mathcal {B}}_{{\underline{n}}}U\ \ \ \ \text { (n-reach tube)}\\ X_{{\underline{n}}}^{\sharp }&=\varvec{A}^{{\underline{n}}}X_0\oplus {\mathcal {B}}_{{\underline{n}}}U\ \ \ \ \text { (n-reach set)}\\ {\hat{X}}_{{\overline{n}}\ \mid \ {\underline{n}}}^{\sharp }&=\tau \left( {\mathcal {A}}^{{\overline{n}}-{\underline{n}}-1}X_{{\underline{n}}}^{\sharp }\oplus {\mathcal {B}}_{{\underline{n}}}U\cap G, U\right) \\ {\hat{X}}_{{\overline{n}}}^{\sharp }&={\hat{X}}_{{\overline{n}}\ \mid \ {\underline{n}}}^{\sharp } \cup {\hat{X}}_{{\underline{n}}}^{\sharp }. \end{aligned}$$

This double step prevents the set $\left\{ \varvec{x} : \varvec{x} \in {\hat{X}}_{{\underline{n}}}^{\sharp }, \varvec{x} \notin X_{{\underline{n}}}^{\sharp } \right\}$ from being included in further computations, thus improving the precision of the over-approximation.

Computing the maximum $\underline{n_i}$ such that Eq. (28) is satisfied is not trivial, because the unknown $\underline{n_i}$ occurs in the exponent of the equation. However, since an intersection with the guard set will always return a sound over-approximation, we do not need a precise value: we can over-approximate it by decomposing $\varvec{g}_i$ into the generalised eigenspace of $\varvec{A}$. More precisely, let

30 $$\begin{aligned} \varvec{g}_i=\sum _{j=1}^p k_{ij} \varvec{v}_j + res (\varvec{g}_i), \end{aligned}$$

where $\varvec{v}_j$ are row vectors of $\varvec{S}^{-1}$ or $-\varvec{S}^{-1}$ such that $k_{ij} \ge 0$, and $res (\varvec{g}_i)$ is the component of the vector $\varvec{g}_i$ that lies outside the range of $\varvec{S}$, namely the subspace spanned by its columns. Notice that since by definition $\varvec{S}$ always has an inverse, it is full rank and therefore $res (\varvec{g}_i)=\varvec{0}$ and subsequently not relevant. It is also important to note that $\varvec{S}$ is the matrix of the generalised eigenvectors of $\varvec{A}$, and that we are therefore expressing the guard in the generalised eigenspace of $\varvec{A}$. Thus, we obtain:

$$\begin{aligned} \rho _{X_0}({\varvec{A}^n}^\intercal \varvec{g}_i) = \rho _{X_0}\left( \sum _{j=1}^p k_{ij} {\varvec{A}^n}^\intercal \varvec{v}_j\right) \le \sum _{j=1}^p k_{ij} \rho _{X_0}\left( {\varvec{A}^n}^\intercal \varvec{v}_j\right) . \end{aligned}$$

### Overestimating the Number of Iterations of a Model Without Inputs

Since rotating dynamics and Jordan shapes will have a complex effect on the behaviour of the model, we seek to transform the Jordan form into a real positive matrix by using the absolute value of the eigenvalues. In such a case, the support function in each direction is monotonically increasing (or decreasing), and it is therefore very easy to find a bound for its progression. We note that the envelope (described by the absolute value) of rotating dynamics will always contain the true dynamics and is therefore a sound over-approximation. We will initially assume that $\gamma _i$ is positive and then extend to the general case.

Let $\rho _{X_0}({\varvec{A}^n}^\intercal \varvec{g}_i)=\rho _{X_0'}({\varvec{J}^n}^\intercal \varvec{g}_i')$, so that $\varvec{g}_i'=\varvec{S}^{-1}\varvec{g}_i$ and

$$\begin{aligned} X_0=\{ \varvec{x} \mid \varvec{C}_{X_0}\varvec{x} \le \varvec{d}_{X_0} \},\quad X_0'=\varvec{S}^{-1}X_0=\{ \varvec{x} \mid \varvec{C}_{X_0}\varvec{S}\varvec{x} \le \varvec{d}_{X_0} \}. \end{aligned}$$

Further, let $\varLambda _\sigma =\{\lambda _i : i\in [1,\ldots ,p],\ \bigwedge _{j=1}^{i-1} (\lambda _i^*\ne \lambda _j \wedge \lambda _i\ne \lambda _j)\}$, be the set of eigenvalues with distinct values (excluding conjugate pairs and geometric multiplicities). Introduce $f_\sigma (\varvec{v}):\mathbb {R}^p \rightarrow \mathbb {R}^{p_b}$, where $p_b$ is the cardinality of $\varLambda _\sigma$, such that $f_\sigma (\varvec{v}) = \text {red}(\varvec{v}_\sigma )$, and

$$\begin{aligned} ({\varvec{v}_\sigma })_i&= \left\{ \begin{array}{ll} 0 &{}\quad \lambda _i \notin \varLambda _\sigma \\ \sqrt{\sum \limits _{j\in [1,\ldots ,p]\wedge (\lambda _j=\lambda _i \vee \lambda _j=\lambda _i^*) }{\varvec{v}_j^2 }}&{}\quad \lambda _i \in \varLambda _\sigma \end{array} \right. , \end{aligned}$$

and furthermore let $F_\sigma : \mathbb {R}^{r \times p} \rightarrow \mathbb {R}^{r \times p_b}$ be

$$\begin{aligned} F_\sigma (\varvec{C})=\varvec{C}_\sigma , \text { where } ({\varvec{C}_\sigma })_{i,*}=f_\sigma (\varvec{C}_{i,*}). \end{aligned}$$

Above, red($\cdot$) is a function that reduces the dimension *p* of a vector to $p_b$ by removing the elements $\lambda _i \notin \varLambda _\sigma$. This reduction is not strictly necessary, but it enables a faster implementation. Thus, given $\varvec{J} = diag\left( \varvec{J}_s, s \in [1,\ldots ,p_b]\right)$, we have

31 $$\begin{aligned} \varvec{J}_\sigma&=\left[ \begin{array}{llll}{\overline{\sigma }}_1&{}\quad 0&{}\quad \cdots &{}\quad 0\\ 0&{}\quad {\overline{\sigma }}_2&{}\quad \cdots &{}\quad 0\\ 0&{}\quad 0&{}\quad \ddots &{}\quad 0\\ 0&{}\quad 0&{}\quad \cdots &{}\quad {\overline{\sigma }}_r\\ \end{array}\right] , \end{aligned}$$

where ${\overline{\sigma }}_s=\sup \limits _{\varvec{x} \ne 0}\frac{\Vert \varvec{J}_s\varvec{x} \Vert _2}{\Vert \varvec{x} \Vert _2}$ is the maximum singular value [48] of the Jordan block $\varvec{J}_s$. Finally, let

32 $$\begin{aligned} \varvec{x}_c'&=\frac{1}{2}(\rho _{X_0'}(\varvec{v_i})+\rho _{X_0'}(-\varvec{v_i})), \quad {\varvec{v}_i}_j= \left\{ \begin{array}{ll} 1 &{}\quad j=i\\ 0 &{}\quad j \ne i \end{array} \right. , \nonumber \\ X_{\sigma }'&= \{\varvec{x} \mid F_\sigma (\varvec{C}_{X_0}\varvec{S})f_\sigma (\varvec{x}) \le f_\sigma (\varvec{d}_{X_0}-\varvec{S}\varvec{C}_{X_0}\varvec{x}_c') \},\nonumber \\ X_0'&\subseteq f_\sigma ^{-1}(X_{c\sigma }'), \text { where } X_{c\sigma }' =\{f_\sigma (\varvec{x}_c')\} \oplus X_{\sigma }' \end{aligned}$$

and $\varvec{v}_\sigma =f_\sigma (\varvec{v})$, where $\varvec{x}_c'$ is the Cartesian centre of $X_0'$ and $X_{c\sigma }'$, an over-approximation of $X_0'$ centred at $\varvec{x}_c'$.

Using properties of eigenvalues and of singular values, we obtain $\rho _{X_0}((\varvec{A}^n)^\intercal \varvec{v}_j) \le {\overline{\sigma }}_j^{\ n}\,\rho _{X_{c\sigma }}((\varvec{v}_\sigma )_j)$, where $j\in [1,\ldots ,p_b]$, and therefore

33 $$\begin{aligned} \rho _{X_0}((\varvec{A}^n)^\intercal \varvec{g}_i) \le \sum _{j=1}^{p_b} k_{ij} {\overline{\sigma }}_j^n\,\rho _{X_{c\sigma }}((\varvec{v}_\sigma )_j), \end{aligned}$$

where $k_{ij}$ are the coefficients in Eq. (30).

Since we have assumed to have no inputs, $\rho _{U_c^n}(\varvec{g}_i) + \rho _{U_b^n}(\varvec{g}_i)=0$, hence we may solve for ${\underline{n}}_i$ as:

34 $$\begin{aligned} \rho _{X_0}((\varvec{A}^{{\underline{n}}_i})^\intercal \varvec{g}_i)&\le \sum _{j=1}^{p_b} k_{ij} {\overline{\sigma }}_j^{\ {\underline{n}}_i}\,\rho _{X_{c\sigma }}((\varvec{v}_\sigma )_j) \le \gamma _i. \end{aligned}$$

In order to separate the divergent parts of the dynamics from the convergent one, let us define

$$\begin{aligned} {\overline{k}}_{ij}=\max \{ k_{ij}\ \rho _{X_{c\sigma }}((\varvec{v}_\sigma )_j), 0 \},\quad {\overline{\sigma }}=\max \{ {\overline{\sigma }}_s : s \in [1,\ldots ,p_b] \}. \end{aligned}$$

This step will allow us to effectively track which trajectories are likely to hit the guard and when, since it is only the divergent element of the dynamics that can increase the size of the reach tube in a given direction. This condition requires that the set of initial conditions is also inside the guard, which is a reasonable assumption.

Substituting in Eq. (34), we obtain

35 $$\begin{aligned} {\overline{\sigma }}^{\ n}\sum _{j=1}^p {\overline{k}}_{ij}\left( \frac{ {\overline{\sigma }}_j}{ {\overline{\sigma }}}\right) ^n \le \gamma _i\;, \end{aligned}$$

which allows us to finally formulate the following iteration scheme for under-approximating *n*.

#### Proposition 1

An iterative under-approximation of the number of iterations *n* can be computed by starting with $\underline{n_i}=1,$ and iterating over

36 $$\begin{aligned} \underline{n_i} \ge n= \log _{ {\overline{\sigma }}}\left( {\gamma _i}\right) -\log _{ {\overline{\sigma }}}\left( {\sum _{j=1}^{p_b} {\overline{k}}_{ij}\left( \frac{ {\overline{\sigma }}_j}{ {\overline{\sigma }}}\right) ^{\underline{n_i}}}\right) \;, \end{aligned}$$

substituting $n_i=n$ on the right-hand side until we meet the inequality. (If $n<0$ is obtained at the first iteration, then ${\underline{n}}_i=0$ is the obtained solution.)

#### Proof

Notice that the sequence $\underline{n_i}$ is monotonically increasing, before it breaks the inequality. As such, any local minimum represents a sound under-approximation of the number of loop iterations. Note that in the case where $\gamma _i \le 0$, we must first translate the system coordinates such that $\gamma _i > 0$. This is simply done by replacing $\varvec{x}'=\varvec{x}+\varvec{c}$ and operating over the resulting system where $\gamma _i' = \rho _{\varvec{c}}(\varvec{g}_i)+\gamma _i$.

Mathematically this is achieved as follows: first we get $\varvec{c}$ by finding the centre of the interval hull (see Eq. (20)) of *G*.^4^ Next we transform the dynamics into

$$\begin{aligned} \left[ \begin{array}{c}\varvec{x}_{k}\\ \varvec{1}\end{array}\right] {=} \left[ \begin{array}{cc}\varvec{A}&{}\varvec{A}\varvec{c}\\ \varvec{0}&{}\varvec{1}\end{array}\right] \left[ \begin{array}{c}\varvec{x}_{k-1}\\ \varvec{1}\end{array}\right] {+}\left[ \begin{array}{c}\varvec{B}\\ \varvec{0}\end{array}\right] \varvec{u}_{k}, \text { where } \left[ \begin{array}{c}\varvec{x}_{k-1}\\ \varvec{1}\end{array}\right] \in \left\{ \left[ \begin{array}{c}\varvec{x}\\ \varvec{1}\end{array}\right] {:} \left[ \begin{array}{cc}\varvec{G}&{}\varvec{G}\varvec{c}\\ \varvec{0}&{}\varvec{1}\end{array}\right] \left[ \begin{array}{c}\varvec{x}_{k-1}\\ \varvec{1}\end{array}\right] {\le } \left[ \begin{array}{c}\varvec{h}\\ \varvec{1}\end{array}\right] \right\} . \end{aligned}$$

$$\square $$

### Underestimating the Number of Iterations of a Model Without Inputs

In order to apply a similar technique to (29), we must find an equivalent under-approximation. In the case of Eq. (34), the quantities ${\overline{\sigma }}_j$ ensure that the equation diverges faster than the real dynamics, hence the estimation found is an upper bound to the desired iteration. In this case we want the opposite, hence we look for a model where the dynamics diverge slower. It is easy to show that ${\lambda _b}_j=|\lambda _j|$ represents these slower dynamics,

$$\begin{aligned} \rho _{X_0}(-{\varvec{A}^{\overline{n_i}}}^\intercal \varvec{g}_i)&\le \sum _{j=1}^p k_{ij} {\lambda _b}_j^{\ \overline{n_i}}\,\rho _{X_{c\sigma }}(-(\varvec{v}_\sigma )_j) \le -\gamma _i, \end{aligned}$$

which reduces to

37 $$\begin{aligned} {\overline{\sigma }}^{\ n}\sum _{j=1}^p {\underline{k}}_{ij}^-\left( \frac{ {\lambda _b}_j}{ {\overline{\sigma }}}\right) ^n +{\overline{\sigma }}^{\ n}\sum _{j=1}^p {\underline{k}}_{ij}^+ \le -\gamma _i\;, \end{aligned}$$

where $${\underline{k}}_{\ ij}^-=\min \left\{ k_{ij}\ \rho _{X_{c\sigma }}(-(\varvec{v}_\sigma )_j)\ ,\ 0 \right\} $$ and $${\underline{k}}_{\ ij}^+=\max \left\{ k_{ij}\ \rho _{X_{c\sigma }}(-(\varvec{v}_\sigma )_j)\ ,\ 0 \right\} $$.

An additional consideration must be made regarding the rotational dynamics. In the previous case we did not care about the rotational alignment of the set $X_n$ with respect to the vector $\varvec{g}_i$, because any rotation would remain inside the envelope corresponding to the absolute value ($r^k cos( k \theta ) \le r^k$ ). In the case of an under-approximation, although the magnitude of a complex eigenvalue at a given iteration may be greater than the support of the guard under verification, its angle with respect to the normal to the support vector may cause the corresponding point to remain inside the guard. We must therefore find iterations that are aligned with the normal to the guard, thus ensuring that the chosen point is outside it. In order to do this, let us first fix the magnitudes of the powered eigenvalues. In the case of convergent dynamics we will assume they have converged a full rotation to make our equation strictly divergent. Let ${\underline{\theta }}=\min \{\theta _j, j \in [1,\ldots ,p]\}$, where $\theta _j$ are the angles of the complex conjugate eigenvalues. Let $n_\theta =\frac{2\pi }{{\underline{\theta }}}$ be the maximum number of iterations needed for any of the dynamics to complete a full turn. Then at any given turn $|\lambda _j|^{{\overline{n}}_i+n_\theta } \le |\lambda _j|^{{\overline{n}}_i+n}, \text { where } |\lambda _i| \le 1 \text { and } n \in [0, n_\theta ]$. This means that any bound we find on the iterations will be necessarily smaller than the true value. Our problem becomes the solution to:

$$\begin{aligned}&\max _n\left( {\overline{\sigma }}^{\ {\overline{n}}_i}\sum _{j=1}^p c_{ij} \cos ((n-{\overline{n}}_i) \theta _j - \alpha _{ij}) \right) ,\quad \alpha _{ij}=\cos ^{-1}(\varvec{g}_i \cdot \varvec{v}_j),\\&c_{ij} = \left\{ \begin{array}{ll} {\underline{k}}_{ij}^-\left( \frac{ {\lambda _b}_j}{ {\overline{\sigma }}}\right) ^{{\overline{n}}_i}&{}|\lambda _j| \ge 1 \\ {\underline{k}}_{ij}^-\left( \frac{ {\lambda _b}_j}{ {\overline{\sigma }}}\right) ^{{\overline{n}}_i+n_\theta }&{}|\lambda _j| < 1 \end{array} \right. . \end{aligned}$$

The problem is simplified by soundly under-approximating the cosines and removing the constants, namely deriving the expressions

$$\begin{aligned}&\max _n\left( {\overline{\sigma }}^{\ {\overline{n}}_i}\sum _{j=1}^p c_{ij} \left( 1-\frac{((n-{\overline{n}}_i) \theta _j - \alpha _{ij})^2}{2}\right) \right) ,\\&\min _n\left( \sum _{j=1}^p c_{ij} {((n-{\overline{n}}_i) \theta _j - \alpha _{ij})^2} \right) ,\text { and } \\&\min _n\left( \sum _{j=1}^p c_{ij}\theta _j^2(n-{\overline{n}}_i)^2 + c_{ij}\alpha _{ij}\theta _j (n-{\overline{n}}_i) \right) . \end{aligned}$$

The solution to the last equation is

38 $$\begin{aligned} n={\overline{n}}_i-\frac{\displaystyle \sum \nolimits _{j=1}^p c_{ij}\alpha _{ij}\theta _j}{\displaystyle 2\sum \nolimits _{j=1}^p c_{ij}\theta _j^2}, \text { with } n \in [{\overline{n}}_i, {\overline{n}}_i+n_\theta ]. \end{aligned}$$

The second part of the equation is expected to be a positive value. When this is not the case, the dominating dynamics will have a rotation $\theta _j \ge \frac{\pi }{2}$. In such cases, we must explicitly evaluate the set up to $\frac{2\pi }{\theta _j}+1$ iterations after ${\overline{n}}_i$, in order to ensure that we have covered a full rotation. If the resulting bound does not satisfy the original inequality: $\rho _{X_0}\left( (\varvec{A}^{\overline{n_i}})^\intercal \varvec{g}_i\right) \ge \gamma _i$, we replace ${\overline{n}}_i=n$ until it does.^5^

#### Proposition 2

An iterative under-approximation of the number of iterations *n* can be computed by starting with $\overline{n_i}'=0$ and iterating over

39 $$\begin{aligned} \overline{n_i}'&\le n= \log _{ {\overline{\sigma }}}\left( {\gamma _i}\right) -\log _{ {\overline{\sigma }}}\left( {\sum _{j=1}^p {\underline{k}}_{ij}^-\left( \frac{ {\lambda _b}_j}{ {\overline{\sigma }}}\right) ^{\overline{n_i}'}}+\sum \limits _{j=1}^p {\underline{k}}_{ij}^+\right) ,\nonumber \\ \overline{n_i}&=\overline{n_i}'+k \text {, given } \rho _{X_0}\left( (\varvec{A}^{(\overline{n_i}'+k)})^\intercal \varvec{g}_i\right) \ge \gamma _i, \end{aligned}$$

where *k* is the result of Eq. (38). We substitute for ${\overline{n}}_i=n$ on the right-hand side as long as the first inequality holds, and then we can find a *k* such that the second inequality holds.

Since we are explicitly verifying the inequality, there is no further proof required.

### Estimating the Number of Iterations of a Model with Inputs

For an LTI model with inputs, we will use the same paradigm explained in the previous section after transforming the system with inputs into an over-approximating system without inputs.

Let $X_{c\sigma }', U_{c\sigma }'$ be the corresponding sets of initial states and inputs obtained by applying Eq. (32) to $X_0'$ and $U_J'$, and let $U_{J\sigma }'=(\varvec{I}-\varvec{J}_\sigma )^{-1} U_{c\sigma }'$. The accelerated resulting system may be represented by the equations

40 $$\begin{aligned} (X_{c\sigma }')_n&=\varvec{J}_\sigma ^n X_{c\sigma }' \oplus (\varvec{I}-\varvec{J}_\sigma ^n)U_{J\sigma '},\nonumber \\ \rho _{(X_{c\sigma }')_n}(\varvec{v})&=\rho _{X_{c\sigma }'}\left( \varvec{J}_\sigma ^{nT}\varvec{v}\right) +\rho _{U_{J\sigma }'}(\varvec{v}) -\rho _{U_{J\sigma }'}\left( \varvec{J}_\sigma ^{nT}\varvec{v}\right) . \end{aligned}$$

Let us now define $(XU)_{\sigma }=\{\varvec{x}-\varvec{u} \mid \varvec{x} \in X_{c\sigma }', \varvec{u} \in U_{J\sigma }' \}$, which allows us to translate the system into

41 $$\begin{aligned} \rho _{((XU)_{\sigma }')_n}(\varvec{v})=\rho _{(XU)_{\sigma }'}\left( \varvec{J}_\sigma ^{nT}\varvec{v}\right) , \end{aligned}$$

which has the same shape as the equations in the previous section. We may now apply the techniques described above to find the bounds on the iterations.

### Narrowing the Estimation of the Number of Iterations

The estimations above can be conservative, but we may obtain tighter bounds on the number of iterations. In the first instance, note that we have eliminated all negative terms in the sums in Eq. (36). Reinstating these terms can result in loss of monotonicity, but we may still create an iterative approach by fixing the negative value at intermediate stages. Let ${\underline{n}}_i$ be our existing bound for the time horizon before reaching a guard, and ${\underline{k}}_{{\underline{n}}_i}=\sum _{j=1}^p {\underline{k}}_{ij}\left( \frac{ {\overline{\sigma }}_j}{ {\overline{\sigma }}}\right) ^{{\underline{n}}_i}$, ${\overline{k}}_{{\underline{n}}_i}=\sum _{j=1}^p {\overline{k}}_{ij}\left( \frac{ {\overline{\sigma }}_j}{ {\overline{\sigma }}}\right) ^{{\underline{n}}_i}$ the corresponding negative and positive terms of the equation. We can now find upper and lower bounds for ${\underline{n}}_i$ by replacing them in Eq. (39) as:

42 $$\begin{aligned} \underline{n_i} \ge n_k=\log _{ {\overline{\sigma }}}\left( {\gamma _i}\right) -\log _{{\overline{\sigma }}}\left( {\overline{k}}_{{\underline{n}}_i}+{\underline{k}}_{{\underline{n}}_k}\right) , \end{aligned}$$

where ${\underline{n}}_k$ is the bound found in the previous stage. Some steps of this process will provide an unsound result, however, every second step will provide a monotonically increasing sound bound which will be tighter than the one in Eq. (36). Since the elements of the sums are convergent, we have that $n_i \ge n_k$ implies ${\underline{k}}_{n_i} \ge {\underline{k}}_{n_k} \left( \text {i.e., }|{\underline{k}}_{n_i}| \le |{\underline{k}}_{n_k}| \right)$, thus

$$\begin{aligned} \log _{{\overline{\sigma }}}\left( {\overline{k}}_{{\underline{n}}_i}+{\underline{k}}_{{\underline{n}}_k}\right) \ge \log _{{\overline{\sigma }}}\left( {\overline{k}}_{{\underline{n}}_i}+{\underline{k}}_{{\underline{n}}_i}\right) , \end{aligned}$$

which means that $n_k$ in Eq. (42) is smaller than our *n* in Eq. (36) ($n_k \le n \le \underline{n_i} \text { and } \underline{n_i} \ge \underline{n_k})$.

In the case of Eq. (39), the explicit evaluation of the guard at each cycle ensures the behaviour described here.

#### Maintaining Geometric Multiplicity

A second step in optimising the number of iterations comes from adding granularity to the bounding abstraction by retaining the geometric multiplicity using the matrix $\varvec{J}_b$ (see Eq. (22)).

##### Lemma 3

Given a matrix $\varvec{A}$ with eigenvalues $\{\lambda _s, s\in [1,\ldots ,r]\}$, where each eigenvalue $\lambda _s$ has a geometric multiplicity $p_s$ and corresponding generalised eigenvectors $\{\varvec{v}_{s,i}, i \in [1,\ldots ,p_s]\}$,

43 $$\begin{aligned} \forall n \ge 0, \varvec{A}^n\varvec{v}_{s}^{i}=\lambda _{s}^n\varvec{v}_{s,i}+\sum _{j=1}^{i-1} \left( {\begin{array}{c}n\\ j\end{array}}\right) \lambda _{s}^{n-j} \varvec{v}_{s,i-j} =\lambda _{s}^n\left( \varvec{v}_{s,i} + \sum _{j=1}^{i-1} \left( {\begin{array}{c}n\\ j\end{array}}\right) \lambda _{s}^{-j}\varvec{v}_{s,i-j}\right) . \end{aligned}$$

##### Proof

By definition, given an eigenvector $\varvec{v}_s$ of $\varvec{A}$, then $\varvec{A}\varvec{v}_s=\lambda _s\varvec{v}_s$ [42]. Similarly, a generalised eigenvector $\varvec{v}_{s,i}$ of $\varvec{A}$ satisfies the equation $\left( \varvec{A}-\lambda _s\varvec{I}\right) \varvec{v}_{s,i}=\varvec{v}_{s,i-1}$^6^. and $\varvec{v}_{s,1}=\varvec{v}_s$, hence

$$\begin{aligned} \varvec{A}\varvec{v}_{s,i}&=\lambda _s\varvec{v}_{s,i}+\varvec{v}_{s,i-1}\\ \varvec{A}^n\varvec{v}_{s,1}&=\lambda _s^n\varvec{v}_{s,1}\\ \varvec{A}^n\varvec{v}_{s,i}&=\varvec{A}^{n-1}(\lambda _s\varvec{v}_{s,i}+\varvec{v}_{s,i-1})=\lambda _s\varvec{A}^{n-1}\varvec{v}_{s,i}+\varvec{A}^{n-1}\varvec{v}_{s,i-1}\\&=\lambda _s^2\varvec{A}^{n-2}\varvec{v}_{s,i}+\lambda _s\varvec{A}^{n-2}\varvec{v}_{s,i-1}+\varvec{A}^{n-1}\varvec{v}_{s,i-1} =\cdots =\lambda _s^n\varvec{v}_{s,i}+\sum _{j=0}^{n-1}\lambda _s^j\varvec{A}^{n-j-1}\varvec{v}_{s,i-1}. \end{aligned}$$

From here we recursively expand the formula for $\varvec{A}^{n-j-1}\varvec{v}_{s,i-1}$ and obtain:

$$\begin{aligned} \varvec{A}^n\varvec{v}_{s,i}&=\lambda _s^n\varvec{v}_{s,i}+\sum _{j=0}^{n-1}\lambda _s^j\lambda _s^{n-j-1}\varvec{v}_{s,i-1}+\sum _{j=0}^{n-1}\sum _{k=0}^{n-2}\lambda _s^k\varvec{A}^{n-k-2}\varvec{v}_{s,i-2}\\&=\lambda _s^n\varvec{v}_{s,i}+n\lambda _s^{n-1}\varvec{v}_{s,i-1}+n\sum _{j=0}^{n-2}\lambda _s^j\varvec{A}^{n-j-2}\varvec{v}_{s,i-2} =\cdots =\lambda _{s}^n\varvec{v}_{s,i}+\sum _{j=1}^{i-1} \left( {\begin{array}{c}n\\ j\end{array}}\right) \lambda _{s}^{n-j}\varvec{v}_{s,i-j}. \quad \square \end{aligned}$$

$$\square $$

Let $i'$ denote the position of $f_b(\lambda _{j})$ within the block $\varvec{J}_{bs}$ it belongs to, such that its corresponding generalised eigenvector is identified as $\varvec{v}_{bs,i'}=f_b(\varvec{v}_j)$. Then

44 $$\begin{aligned} \rho _{X_0'}({\varvec{J}^n}^\intercal \varvec{g}_i')&\le \sum _{j=1}^{p_b} k_{ij} \rho _{X_0}\left( {\varvec{J}_b^n}^\intercal f_b(\varvec{v}_j)\right) \le \sum _{j=1}^{p_b} k_{ij} {\lambda _b}_j^n \rho _{X_0}\left( \varvec{v}_{bs,i'} + \sum _{k=1}^{i'-1} \left( {\begin{array}{c}n\\ k\end{array}}\right) {\lambda _b}_j^{-k}\varvec{v}_{bs,i'-k}\right) \nonumber \\&\le \sum _{j=1}^{p_b} k_{ij} {\lambda _b}_j^n \left( \rho _{X_0}\left( \varvec{v}_{bs,i'}\right) + \sum _{k=1}^{i'-1} \left( {\begin{array}{c}n\\ k\end{array}}\right) {\lambda _b}_j^{-k}\rho _{X_0}\left( \varvec{v}_{bs,i'-k}\right) \right) \nonumber \\&\le \sum _{j=1}^{p_b} k_{ij0}' {\lambda _b}_j^n+ \sum _{m=1}^{i'} k_{ijm}' {\lambda _b}_j^n\prod \limits _{m=0}^{p_s-i'-1} (n-m). \end{aligned}$$

In order to manage the product on the right hand side we use slightly different techniques for over- and under-approximations. For ${\underline{n}}_i$ we first find an upper bound ${\underline{n}}_i'$ using Eq. (36) and $k_{ij}={k_{ij}'}_0+{k_{ij}'}_m$, and then do a second iteration using $k_{ij}={k_{ij}'}_0+{k_{ij}'}_m\prod \nolimits _{m=0}^{p_s-i'-1} ({\underline{n}}_i'-m)$, which ensures that the true value is below the approximation. In the case of ${\overline{n}}_i$, we also start with $k_{ij}={k_{ij}'}_0+{k_{ij}'}_m$ and update it during the iterative process.

##### Example 2

Let us look at the following example, comprising matrices:

$$\begin{aligned} \varvec{J}={ \left[ \begin{array}{llllll} 3&{}\quad 0&{}\quad 0&{}\quad 0&{}\quad 0&{}\quad 0\\ 0&{}\quad 2&{}\quad 1&{}\quad 0&{}\quad 0&{}\quad 0\\ 0&{}\quad 0&{}\quad 2&{}\quad 0&{}\quad 0&{}\quad 0\\ 0&{}\quad 0&{}\quad 0&{}\quad -2&{}\quad 0&{}\quad 0\\ 0&{}\quad 0&{}\quad 0&{}\quad 0&{}\quad -1&{}\quad 1\\ 0&{}\quad 0&{}\quad 0&{}\quad 0&{}\quad -1&{}\quad -1 \end{array}\right] }\,\text {, }\,\, \varvec{S}={ \left[ \begin{array}{llllll} 1&{}\quad 0&{}\quad 0&{}\quad 0&{}\quad 0&{}\quad 0\\ 0&{}\quad 3&{}\quad 0&{}\quad 0&{}\quad 0&{}\quad 0\\ 0&{}\quad -4&{}\quad 1&{}\quad 0&{}\quad 0&{}\quad 0\\ 0&{}\quad 0&{}\quad 0&{}\quad 1&{}\quad 0&{}\quad 0\\ 0&{}\quad 0&{}\quad 0&{}\quad 0&{}\quad 1&{}\quad 0\\ 0&{}\quad 0&{}\quad 0&{}\quad 0&{}\quad 1&{}\quad 1 \end{array}\right] }\,\text {, }\,\, \varvec{J}_{\sigma }={ \left[ \begin{array}{llll} 3&{}\quad 0&{}\quad 0&{}\quad 0\\ 0&{}\quad 3&{}\quad 0&{}\quad 0\\ 0&{}\quad 0&{}\quad 2&{}\quad 0\\ 0&{}\quad 0&{}\quad 0&{}\quad \sqrt{2} \end{array}\right] }, \end{aligned}$$

with $\varvec{J}_\sigma$ calculated as in Eq. (31), initial condition $$\varvec{x}_0' = {\left[ \begin{array}{llllll}1&\quad 1&\quad 1&\quad 1&\quad 1&\quad 1\end{array}\right] }$$ and guard set $\varvec{G}\varvec{x} \le 300$ where

$$\begin{aligned} \varvec{G}{=\left[ \begin{array}{llllll}1&\quad 3&\quad -3&\quad 2&\quad 4&\quad 1\end{array}\right] } ={\left[ \begin{array}{llllll}1&\quad 1&\quad 1&\quad 2&\quad 4&\quad -3\end{array}\right] }\varvec{S}^\intercal . \end{aligned}$$

The progression of the support function of the reach sets along this vector and the corresponding bounds, as described in the previous section, are shown in Fig. 10.

**Fig. 10:**
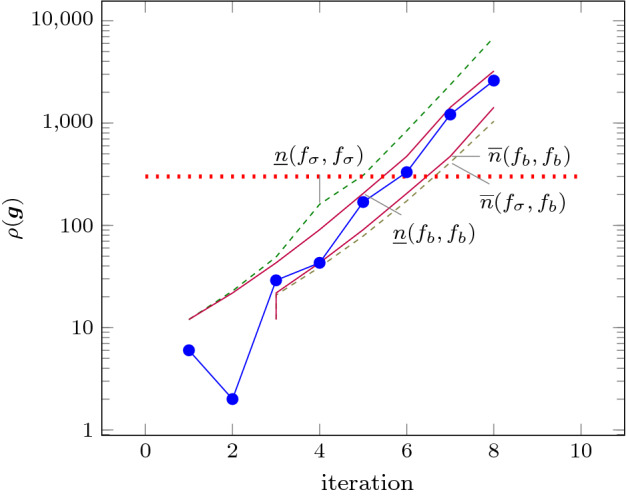
Progression of the support function of a system for a given guard. The thick blue dots are real values. The dashed green line over-approximates the progression using singular values (Sect. 7.1), the dashed yellow line under-approximates them using eigenvalue norms (Sect. 7.2), whereas the continuous purple lines represent the tighter over-approximation maintaining the geometric multiplicity (Sect. 7.4.1). We can see how the purple line finds a better bound for ${\underline{n}}_i$, while the ${\overline{n}}_i$ bound is conservative for both approaches. (Color figure online)

Changing the eigenvalues to:

$$\begin{aligned} \varvec{J}&=\left[ \begin{array}{llllll} 2e^{-0.2i}&{}\quad 0&{}\quad 0&{}\quad 0&{}\quad 0&{}\quad 0\\ 0&{}\quad 2e^{0.2i}&{}\quad 0&{}\quad 0&{}\quad 0&{}\quad 0\\ 0&{}\quad 0&{}\quad \sqrt{2}e^{-0.3i}&{}\quad 0&{}\quad 0&{}\quad 0\\ 0&{}\quad 0&{}\quad 0&{}\quad \sqrt{2}e^{0.3i}&{}\quad 0&{}\quad 0\\ 0&{}\quad 0&{}\quad 0&{}\quad 0&{}\quad 1.1e^{0.5i}&{}\quad 0\\ 0&{}\quad 0&{}\quad 0&{}\quad 0&{}\quad 0&{}\quad 1.1e^{-0.5i} \end{array}\right] , \end{aligned}$$

we obtain the results in Fig. 11. In this second case we can see that the rotational dynamics force an increase of the initially calculated iteration to account for the effects of the rotation. $\square$

**Fig. 11:**
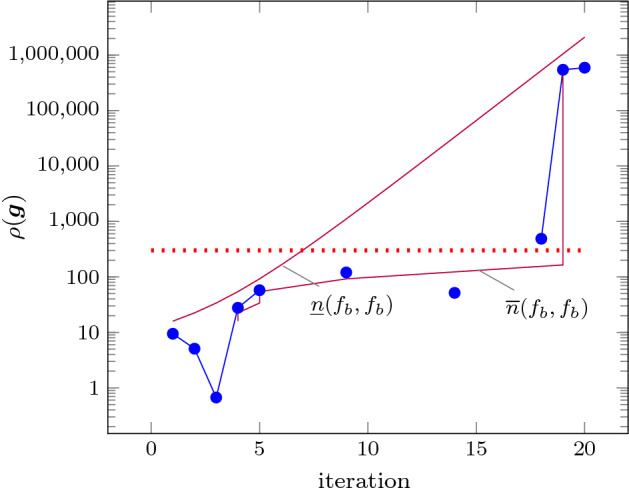
Progression of the support function of a rotational system for a given guard. The thick blue dots are real values (negative values are missing due to the log scale). Continuous purple lines represent the over-approximation. The steep vertical line at iteration 19 is due to the alignment of the rotations with the guard at this point. The point at iteration 14 appears below the line because of the higher point at iteration 9. The procedure will either find that this boundary was met at iteration 9 or push it forward to iteration 19. (Color figure online)

### Case Study

We have selected a known benchmark from the literature to illustrate the discussed procedure: the room temperature control problem [28]. The temperature (variable $temp$) of a room is controlled via a user-defined input ($set$), which can be changed at any discrete time step through a heating ($heat$) element, and is affected by ambient temperature ($amb$) that is out of the control of the model.

We formalise the description of such a system both via a linear loop and with a dynamical model. Observe that since such a system may be software controlled, Algorithm 1 gives a pseudo-code fragment for the temperature control problem.
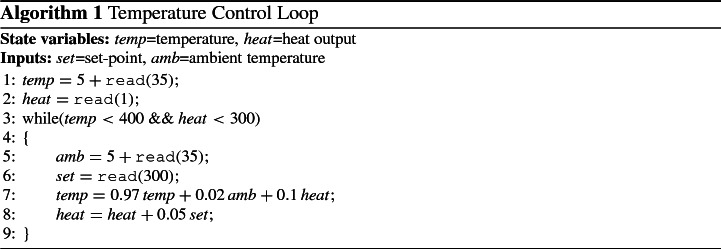
 We use the read function to represent non-deterministic values between 0 and the maximum value given as its argument. Alternatively, this loop corresponds to the following hybrid dynamical model:

$$\begin{aligned} \left[ \begin{array}{c} temp \\ heat \\ \end{array} \right] _{k+1}&= \left[ \begin{array}{ll} 0.97 &{}\quad 0.1\\ -0.05 &{}\quad 1\\ \end{array} \right] \left[ \begin{array}{c} temp \\ heat \\ \end{array} \right] _{k} + \left[ \begin{array}{cc} 0.02 &{}\quad 0\\ 0 &{}\quad 0.05\\ \end{array} \right] \left[ \begin{array}{c} amb \\ set \\ \end{array} \right] _{k}, \end{aligned}$$

with initial condition $$\left[ \begin{array}{c} temp \\ heat \\ \end{array} \right] _{0} \in \left[ \begin{array}{c} \left[ 5, 40 \right] \\ \left[ 0, 1 \right] \\ \end{array} \right] $$, non-deterministic inputs $$ \left[ \begin{array}{c} amb \\ set \\ \end{array} \right] _{k} \in \left[ \begin{array}{c} \left[ 5, 40 \right] \\ \left[ 0, 300 \right] \\ \end{array} \right] , $$ and guard set $$G = \left\{ \left[ \begin{array}{c} temp \\ heat \\ \end{array} \right] : \left[ \begin{array}{ll} 1 &{}\quad 0\\ 0 &{}\quad 1\\ \end{array} \right] \left[ \begin{array}{c} temp \\ heat \\ \end{array} \right] < \left[ \begin{array}{c} 400\\ 300\\ \end{array} \right] \right\} $$.

In this model the variables are continuous and take values over the real line, whereas within the code they are represented as long double precision floating-point values, with precision of $\pm 10^{-19}$, moreover the error of the approximate Jordan form computation results in $\delta _{max}<10^{-17}$. The eigendecomposition of the dynamics is (the values are rounded to three decimal places):

$$\begin{aligned} \varvec{A}&=\varvec{S}\varvec{J}\varvec{S}^{-1} \subseteq \mathbb {S}\mathbb {J}\mathbb {S}^{-1},\text { where }\\ \mathbb {S}&=\left[ \begin{array}{cc} 0.798 \pm 10^{-14} &{} 0.173 \pm 10^{-15}\\ 0 \pm 10^{-19} &{} 0.577\pm 10^{-14}\\ \end{array} \right] ,\\ \mathbb {J}&= \left[ \begin{array}{ll} 0.985 \pm 10^{-16}&{}\quad 0.069 \pm 10^{-17}\\ -0.069 \pm 10^{-17}&{}\quad 0.985 \pm 10^{-16}\\ \end{array} \right] ,\\ \mathbb {S}^{-1}&= \left[ \begin{array}{ll} 1.253 \pm 10^{-12} &{}\quad - 0.376\pm 10^{-13}\\ 0 \pm 10^{-18} &{}\quad 1.732 \pm 10^{-12}\\ \end{array} \right] . \end{aligned}$$

The discussed over-approximations of the reach-sets indicate that the temperature variable intersects the guard set *G* at iteration ${\underline{n}}=32$. Considering the pseudo-eigenvalue matrix along these iterations, we use Eq. (13) to find that the corresponding complex pair remains within the following boundaries:

$$\begin{aligned} \begin{array}{lr} {\mathcal {A}}^{32} = \left[ \begin{array}{ll} r &{}\quad i \\ -i &{}\quad r\\ \end{array} \right] \left\{ \begin{array}{lllll} 0.4144 &{}\quad<&{}\quad r &{}\quad< &{}\quad 0.985\\ 0.0691 &{}\quad<&{}\quad i &{}\quad< &{}\quad 0.7651\\ 0.1082 &{}\quad<&{}\quad r+ i &{}\quad< &{}\quad 1.247\\ 0.9159 &{}\quad<&{}\quad i - r &{}\quad< &{}\quad 0.9389\\ \end{array} \right. \;\;,\;\;\;\; {\mathcal {B}}^{32} = \left[ \begin{array}{ll} r &{}\quad i \\ -i &{}\quad r\\ \end{array} \right] \left\{ \begin{array}{lllll} 1 &{}\quad<&{}\quad r &{}\quad< &{}\quad 13.41\\ 0 &{}\quad<&{}\quad i &{}\quad< &{}\quad 17.98\\ 1 &{}\quad<&{}\quad r+ i &{}\quad< &{}\quad 29.44\\ 6.145 &{}\quad<&{}\quad i - r &{}\quad < &{}\quad 6.514\\ \end{array} \right. . \end{array} \end{aligned}$$

The reach tube is calculated by multiplying these abstract matrices with the initial sets of states and inputs, as described in Eq. (25), by the following inequalities:

$$\begin{aligned} {\hat{X}}_{32}^\#=&{\mathcal {A}}^{32} \left[ \begin{array}{c} \left[ 5, 40 \right] \\ \left[ 0, 1 \right] \\ \end{array} \right] + {\mathcal {B}}^{32} \left[ \begin{array}{c} \left[ 5, 40 \right] \\ \left[ 0, 300 \right] \\ \end{array} \right] = \left[ \begin{array}{c} temp\\ heat\\ \end{array} \right] \left\{ \begin{array}{lllll} -24.76 &{}\quad<&{}\quad temp &{}\quad< &{}\quad 394.5\\ -30.21 &{}\quad<&{}\quad heat &{}\quad< &{}\quad 253\\ -40.85 &{}\quad<&{}\quad temp+ heat &{}\quad< &{}\quad 616.6\\ -86.31 &{}\quad<&{}\quad temp - heat &{}\quad < &{}\quad 843.8\\ \end{array} \right. . \end{aligned}$$

The negative values represent the lack of restriction in the code on the lower side and correspond to a cooling system (negative heating). The set is displayed in Fig. 12, where for the sake of clarity only 8 directions of the 16 constraints are shown. This results in a rather tight over-approximation which, for comparison’s sake, is not looser than the convex hull of all reach sets obtained by [31] using the given directions. Figure 12 further displays the initial set in black, the collection of reach sets in white, the convex hull of all reach sets in dark blue (as computed by [31]), and finally the abstractly accelerated set in light yellow (dashed lines). The outer lines represent the guards for *G*.

**Fig. 12:**
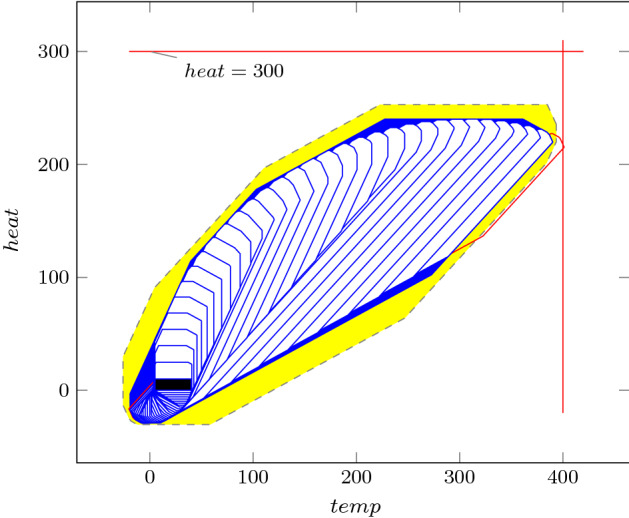
The abstractly accelerated tube (yellow, dashed boundary), representing an over-approximation of the thermostat reach tube (dark blue). The set of initial conditions is shown in black, whereas successive reach sets are shown in white. The guards and the reach set that crosses them are close to the boundary in red. (Color figure online)

## Application of Abstraction-Refinement to Abstract Acceleration

One of the main limitations of abstract acceleration is that, despite being very fast, it leverages an over-approximation of the actual reach tube for verification. In many cases this over-approximation can be too coarse (i.e., imprecise) for the proof of the safety property of interest. This section deals with methods for refining this over-approximation. Refinements are based on counterexamples, namely vertices of the abstract matrix that lay outside the projection of the safety specification onto the abstract space (calculated using the inverse of the reachability transformations). Our approach can be seen as an instance of the known CounterExample Guided Abstraction Refinement (CEGAR) paradigm [20].

### Finding Counterexample Iterations

Because the objective is to refine the abstract dynamics, we need to find the iterations corresponding to the counterexample (i.e., the ones used to calculate the hyperplanes forming the unsafe vertex). This will allow us to find an interpolant iteration that will reduce the polyhedron in the right direction. Since the abstract dynamics are built over pairs of eigenvalues, it is possible that different eigenvalue pairs provide different results for the counterexample iteration, in which case all of them are used. Let a verification clause explore the solution $\rho _{\mathcal {A}}(\varvec{v}) = s \le {\overline{s}}$, where $\varvec{v}$ is the direction we are examining, *s* its corresponding support function, and $\rho _{\mathcal {A}}(\varvec{v}) \le {\overline{s}}$ the safety specification. If $s>{\overline{s}}$ the specification will not be met and we need a refinement. Let $\varvec{a}_v \in {\mathcal {A}}$ be the vertex at which the maximum is found, i.e., $\varvec{a}_v \cdot \varvec{v}=s$. The iterations corresponding to this counterexample may be found by analysing the dynamics of each pair of eigenvalues independently, and finding the point closest to the hyperplane whose inequality has been violated. This is done as follows:

1. *Conjugate Eigenvalues* Since the trajectories along these are circular and centred at the origin, we can find the angle that $\varvec{a}_v$ forms with the axes of the eigenvalues and use it to calculate the right iteration. Let $\theta _i$ be the angle of the conjugate eigenvalue pair and $\theta _{\varvec{a}_v}(i)$ the angle formed by $\varvec{a}_v$ in the $i{\text {th}}$ plane (this is equivalent to $\tan ^{-1}\left( \frac{(\varvec{a}_v)_i}{(\varvec{a}_v)_{i+1}}\right)$). The corresponding iteration will depend on whether the eigenvalue is convergent or divergent. In the former case, it will be $\frac{\theta _{\varvec{a}_v}(i)}{\theta _i}$, and in the latter it will be $(n - (n \mod \frac{1}{\theta _i}))+\frac{\theta _{\varvec{a}_v}(i)}{\theta _i}$, where $\hbox {mod}$ is the modulus operation over the reals.
2. *Real Eigenvalues* In the case of reals, finding the iteration relies on the direct relation between the given eigenvalue and the target counterexample. Since $(\varvec{a}_v)_i \approx \lambda _i^k \Rightarrow k \approx \log _{\lambda _i}((\varvec{a}_v)_i)$. If the logarithm does not exist, then we presume we cannot further refine using this method.
3. *Jordan Blocks with Non-unitary Geometric Multiplicity* In the case of larger Jordan blocks we need to examine the nature of the dynamics. Let us look at the equation representing the contribution of a Jordan block to the support:
  45 $$\begin{aligned} \rho _{\lambda _s}(\varvec{v})=\sum _{j=0}^{p_s} \left( {\begin{array}{c}n\\ j\end{array}}\right) \lambda ^{n-j} {\varvec{v}_s}_j. \end{aligned}$$
  In this case we must use an iterative approximation, as described in Sect. 7.4.1, to find the closest iteration to the unsafe guard. Although this process is more costly than the ones described above, it is also more precise, thus providing a much better refinement. Note that the technique can be applied to the full set of eigenvalues or to any subset of Jordan blocks. This choice is a compromise between precision and speed. We also note that when the refinement process is done in the eigenspace, the new eigenvectors are now the identity set, which makes the problem more tractable.

Since the exclusion of an unsafe vertex from the abstract dynamics does not ensure a sufficiently tight over-approximation, we must perform this step iteratively until either we run out of new refinements or reach a user-defined timeout.

Once the candidate iterations are found, it suffices to add further constraints to the abstract matrix for these iterations as described in Fig. 13. Notice that given the above procedure, it is often faster and more beneficial to begin by performing the refinement over the complex eigenvalues by directly examining the vector of directions $\varvec{v}$ in the corresponding sub-spaces.

**Fig. 13:**
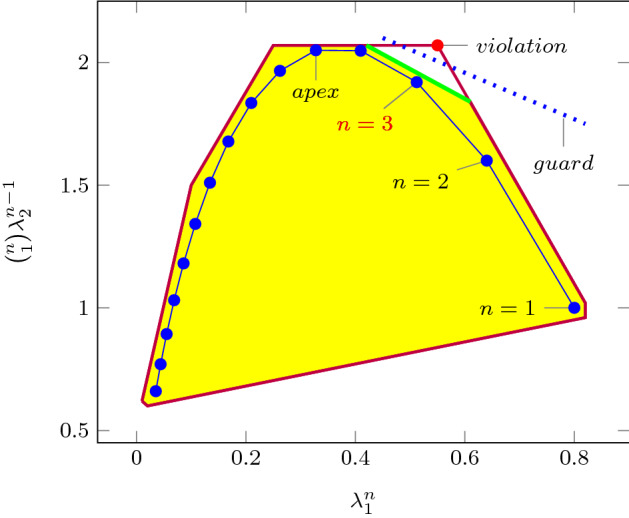
Polyhedral faces from an $\mathbb {R}^2$ Jordan block subspace $$(\lambda _1^n, \left( {\begin{array}{c}n\\ 1\end{array}}\right) \lambda _2^{n-1})$$ where $\lambda _1=0.8, \lambda _2=0.8$ and $1 \le n \le 15$. The red dot specifies an abstract vertex violating the safety specification (dashed blue line). The closest iteration to the violating vertex is $n=3$. A new support function (green) based on $n=3$ eliminates the violating vertex. The new abstract polyhedron meets the safety specification (yellow). (Color figure online)

### Using Bounded Concrete Runs

Owing to the complex interactions in the dynamics, the above procedure may not always find the correct iterations for refinement, or at least not optimal ones. For this reason, a second method is proposed, which in most cases will be more efficient and precise when the dynamics are strictly convergent.

This second approach relies on the direct calculation of the initial ${\overline{k}}$ iterations. Since we operate over the eigenvalues and we limit ${\overline{k}}$ to a conservative bound, this is a relatively inexpensive calculation. The approach leverages the idea that for convergent dynamics, counterexamples are often found in the initial runs. The first step is to directly calculate the trajectory of the counterexample for the first ${\overline{k}}$ iterations, and its corresponding support function in the direction of $\varvec{v}$. Once again, because this is a single point and a bounded time, this operation is comparatively inexpensive. The second step consists of finding an upper bound for all subsequent iterations, which we can do by using the norms of the eigenvalues and the peaks of each geometrical multiple of a Jordan Block (which relate to these norms). By selecting the larger between these two supports, we ensure soundness over the infinite time horizon. This is equivalent to evaluating the reach tube as

$$\begin{aligned} X_n^\sharp =\bigcup \limits _{k=0}^{{\overline{k}}} \varvec{A}^kX_0 \cup {\mathcal {A}}_{n-{\overline{k}}}\varvec{A}^{{\overline{k}}}X_0 \;. \end{aligned}$$

Since the result above is known to be an upper bound for the support in the direction of $\varvec{v}$, we can directly add it to the inequalities of ${\mathcal {A}}$.

### Case Study

We have taken an industrial benchmark ‘CAFF problem Instance: Building’.^7^ The benchmark consists of a continuous model with 48 state variables and one input. Furthermore, there is an initial state corresponding to a 10-dimensional hyper-rectangle. Time is discretised using a 5 ms sample interval to give us a discrete time model for verification. Notice that the choice of sample time has very little effect on abstract acceleration. It mainly affects the requirement for floating point precision (as very small angles may require higher precision), and may have an effect on counterexample generation which can either decrease precision or increase time (i.e., we may relax the precision of the algorithm to gain speed or tightening at the cost of higher computation times). The provided model requires an analysis on the $25{\text {th}}$ variable, with a safety specification requiring it to remain below .005. The problem has been verified using SpaceEx^8^ for bounded time (20 s) in under three seconds. Axelerator was run on this benchmark using different parameters. We used an Intel 2.6 GHz i7 processor with 8 GB of RAM running on Linux. Although the algorithm lends itself to concurrency, the tool currently supports only single threading. The process itself uses 82 MB on this particular benchmark. The results are summarised in Table 1. Since many tools in this area use unsound arithmetic, we present results for both sound and unsound arithmetic using abstract acceleration to quantify the cost of soundness. It is worth noting that for precisions under 1024 bits the tool returns soundness violation errors when using sound arithmetic.

We note in these results that the performance of Axelerator depends largely on the required level of refinement. State of the art tools can do bounded model checking faster than Axelerator, largely due to implementation optimisations (we expect a better simplex engine would allow us to be more competitive in this regard). This advantage disappears as soon as we require a larger time horizon for verification.

**Table 1:** Axelerator performance on 48 dimensional building benchmark using various settings

Refinement	Guard	Sound	Inputs	Bits	Time (s)	Bound
No refinement	.015	No	V	80	9	0.013693
Refinement	.005	No	V	80	18	0.004996
No refinement	.015	No	P	128	13	0.013633
No refinement	.015	No	V	128	24	0.013716
Refinement	.005	No	P	128	29	0.004996
Refinement	.005	No	V	128	48	0.004976
Refinement	.005	No	P	1024	66	0.004996
Refinement	.005	No	V	1024	90	0.004999
Refinement	.005	Yes	P	1024	190	0.004996
Refinement	.005	Yes	V	1024	563	0.004998

P = Parametric, V = Time-varying. We see that in this case, the refinement phase doubles the analysis time. We also show the results of Counterexample Refined Abstract Acceleration for different types of inputs, bit lengths and soundness

## Experimental Results

The overall Abstract Acceleration procedure has been implemented in C++ using the eigen-algebra package (v3.2), with multiple precision floating-point arithmetic, and has been tested on a 1.6 GHz Core 2 Duo computer. Unless otherwise specified, we use the sound version of Abstract Acceleration without abstraction-refinement (as per Sect. 8). The tool, called *Axelerator*, and the corresponding benchmarks used to test it (including specifications of the initial states, input ranges and guard sets), are available at

http://www.cprover.org/LTI/

Since many tools require the specification of a set of directions in which to perform verification, we have chosen to use an octahedral template set. That is the set of vectors that run either along an axis or at a 45 degree angle between two axes (equivalent to a set of inequalities $\pm x_i \le bound_k$ or $\pm x_i \pm x_j<bound_k$). We also make use of interval hulls, which are defined as the smallest hyper-boxes that enclose a set (equivalent to $\pm x_i \le bound_k$).

**Table 2:** Experimental comparison of unbounded-time analysis tools with inputs

Name	Characteristics				new bounds		analysis time (sec)		
	Type	Dim	Inputs	Bounds	IProc	Sting	IProc	Sting	mpfr
parabola_i1	$\lnot s$,$\lnot c$,*g*	2	1	80	+ 25 (31%)	+ 28 (35%)	0.007	237	0.049
parabola_i2	$\lnot s$,$\lnot c$,*g*	2	1	80	+ 24 (30%)	+ 35 (44%)	0.008	289	0.072
cubic_i1	$\lnot s$,$\lnot c$,*g*	3	1	120	+ 44 (37%)	+ 50 (42%)	0.015	704	0.097
cubic_i2	$\lnot s$,$\lnot c$,*g*	3	1	120	+ 35 (30%)	+ 55 (45%)	0.018	699	0.124
oscillator_i0	*s*,*c*,$\lnot g$	2	0	56	+ 24 (43%)	+ 24 (43%)	0.004	0.990	0.021
oscillator_i1	*s*,*c*,$\lnot g$	2	0	56	+ 24 (43%)	+ 24 (43%)	0.004	1.060	0.024
inv_pendulum	*s*,*c*,$\lnot g$	4	0	16	+ 8 (50%)	+ 8 (50%)	0.009	0.920	0.012
convoyCar2_i0	*s*,*c*,$\lnot g$	3	2	12	+ 9 (75%)	+ 9 (75%)	0.007	0.160	0.043
convoyCar3_i0	*s*,*c*,$\lnot g$	6	2	24	+ 15 (62%)	+ 15 (62%)	0.010	0.235	0.513
convoyCar3_i1	*s*,*c*,$\lnot g$	6	2	24	+ 15 (62%)	+ 15 (62%)	0.024	0.237	0.901
convoyCar3_i2	*s*,*c*,$\lnot g$	6	2	24	+ 15 (62%)	+ 15 (62%)	0.663	0.271	1.416
convoyCar3_i3	*s*,*c*,$\lnot g$	6	2	24	+ 15 (62%)	+ 15 (62%)	0.122	0.283	2.103

**Type**: *s* – stable loops, *c* – complex eigenvalues, *g* – loops with guard; **dim**: model dimension (variables); **bounds**: number of half-planes defining the reach tube; **new bounds**: number of bounds newly detected by Axelerator (mpfr) over the existing tools (IProc, Sting); Axelerator detects all bounds, therefore there are no lost bounds vs. existing tools. **IProc** is [43]; **Sting** is [22]; **mpfr** is Axelerator (this work) using 256 bit mantissa

### Comparison with Other Unbounded-Time Approaches

**Table 3:** Experimental comparison with previous work

Name	Characteristics			Improved		Analysis time (s)					
	Type	Dim	Bounds	Tighter	Looser	J	(jcf)	mpfr+(jcf)		mpfr	ld
parabola_i1	$\lnot s$,$\lnot c$,*g*	3	80	+ 4 (5%)	0 (0%)	2.51	( 2.49)	0.16	(0.06)	0.097	0.007
parabola_i2	$\lnot s$,$\lnot c$,*g*	3	80	+ 4 (5%)	0 (0%)	2.51	( 2.49)	0.26	(0.06)	0.101	0.008
cubic_i1	$\lnot s$,$\lnot c$,*g*	4	120	0 (0%)	0 (0%)	2.47	( 2.39)	0.27	(0.20)	0.110	0.013
cubic_i2	$\lnot s$,$\lnot c$,*g*	4	120	0 (0%)	0 (0%)	2.49	( 2.39)	0.32	(0.20)	0.124	0.014
oscillator_i0	*s*,*c*,$\lnot g$	2	56	0 (0%)	− 1 (2%)	2.53	( 2.52)	0.12	(0.06)	0.063	0.007
oscillator_i1	*s*,*c*,$\lnot g$	2	56	0 (0%)	− 1 (2%)	2.53	( 2.52)	0.12	(0.06)	0.078	0.008
inv_pendulum	*s*,*c*,$\lnot g$	4	12	+ 8 (50%)	0 (0%)	65.78	(65.24)	0.24	(0.13)	0.103	0.012
convoyCar2_i0	*s*,*c*,$\lnot g$	5	12	+ 9 (45%)	0 (0%)	5.46	( 4.69)	3.58	(0.22)	0.258	0.005
convoyCar3_i0	*s*,*c*,$\lnot g$	8	24	+ 10 (31%)	− 2 (6%)	24.62	(11.98)	3.11	(1.01)	0.552	0.051
convoyCar3_i1	*s*,*c*,$\lnot g$	8	24	+ 10 (31%)	− 2 (6%)	23.92	(11.98)	4.94	(1.01)	0.890	0.121
convoyCar3_i2	*s*,*c*,$\lnot g$	8	24	+ 10 (31%)	− 2 (6%)	1717.00	(11.98)	6.81	(1.01)	1.190	0.234
convoyCar3_i3	*s*,*c*,$\lnot g$	8	24	+ 10 (31%)	− 2 (6%)	1569.00	(11.98)	8.67	(1.01)	1.520	0.377

**Type**: *s* stable loops, *c* complex eigenvalues, *g* loops with guard; **dim**: model dimension; **bounds**: no. of half planes defining the reach tube; **improved**: number of bounds (and percentage) that were tighter (better) or looser (worse) than J; **J** is Schrammel et al. [44]; **mpfr+** is this work using 1024 bit mantissa ($e<10^{-152}$); **mpfr** uses a 256 bit mantissa ($e<10^{-44}$); **ld** uses a 64 bit mantissa ($e<10^{-11}$); here *e* is the accumulated error of the dynamical system; **jcf**: time taken to compute the Jordan form

In a first experiment we have benchmarked our implementation against the tools InterProc [43] and Sting [22]. We have tested these tools on different discrete time models, including guarded/unguarded ones, stable/unstable ones, and models with complex/real loops with inputs (details in Table 2). In the first instance, we can see that Axelerator is more successful in finding tight over-approximations that remain very close to the actual reach set. InterProc and Sting are each unable to find nearly 40% of the bounds (i.e., supports), meaning they report an infinite reach-space in the corresponding directions. In these instances, InterProc (owing to limitations related to widening) and Sting (owing to the absence of tight polyhedral, inductive invariants) are unable to infer finite bounds at all. The precision provided by our implementation comes at a reasonable price in terms of computational effort: Axelerator is approximately 10 times slower than InterProc, and in most cases faster than Sting.

Table 3 presents a comparison of our implementation using different levels of precision (long double, 256 bit, and 1024 bit floating-point precision) with the core procedure for abstract acceleration of linear loops without inputs (J) [44] (we use a version without inputs of the parabola, cubic and convoy models). This shows that our implementation gives tighter over-approximations on most benchmarks (column ‘improved’). While on a limited number of instances the current implementation is less precise (the lower right portion of Fig. 4 where the dotted red line crosses into the inside of our support gives a hint why this is happening), the overall increased precision results from mitigating the limitation on chosen directions caused by the use of logahedral abstractions (as done in previous work [44]).

At the same time, our implementation is faster than [44], partly owing to the use of numeric eigendecomposition (as opposed to symbolic), but mostly in view of the cost of increasingly large rational representations in [44] needed to maintain precision when evaluating higher order systems. The fact that many bounds have improved with the new approach, while speed has increased by several orders of magnitude, provides evidence of the advantages of the new approach.

The speed-up is due to the faster Jordan form computation, which takes between 2 and 65 seconds for [44] (using the ATLAS package), whereas our implementation requires at most one second. For the last two benchmarks, the polyhedral computations blow up in [44], whereas our support function approach shows only moderately increasing runtimes. The increase of speed is owing to multiple factors, as detailed in Table 4. The difference in precision of using long double vs. multiple precision floating-point arithmetic is negligible, as all results in the given examples match exactly to 9 decimal places.

**Table 4:** Performance improvements over [44]

Optimisation	Speed-up	
Eigen versus ATLA$^\text {a}$	2	10
Support functions versus octahedral templates	2	40
Long double versus multiple precision arithmetic	5	200
Interval versus regular arithmetic	0.2	0.5
Total	4	80,000

### Comparison with Bounded-Time Approaches

In a third experiment, we compare our method with the LGG algorithm [51] used by SpaceEx [31]. Since LGG provides the tightest supports for a given set of directions, this study indicates the quality of our over-approximation. In order to set up a fair comparison, we have provided the implementation of the native algorithm in [51] within our software. We have run both methods on the convoyCar example [44] with inputs, which presents an unguarded, scalable (the dimension can be increased by adding more cars), stable loop with complex dynamics, and we have focused focused on octahedral templates. For convex reach tubes, the approximations computed by Abstract Acceleration are reasonably tight in comparison to those computed by the LGG algorithm (see Table 5). However, by storing finite disjunctions of convex polyhedra, the LGG algorithm is able to generate non-convex reach tubes, which are arguably tighter in case of oscillating or spiralling dynamics. Still, in many applications abstract acceleration can provide a tight over-approximation for the convex hull of (possibly non-convex) reach tubes.

**Table 5:** Comparison on convoyCar2 benchmark [44], between this work and the LGG algorithm from [51]. The intervals give the minimum and maximum values obtained for each variable

Name	Axelerator		Axelerator using LGG		
	100 iterations	Unbounded	100 iterations	200 iterations	300 iterations
Run time	166 ms	166 ms	50 ms	140 ms	195 ms
Car acceleration	$[-0.820, 1.31]$	$[-1.262, 1.31]$	$[-0.815, 1.31]$	$[-0.968, 1.31]$	$[-0.968, 1.31]$
Car speed	$[-1.013, 5.11]$	$[-4.515, 6.15]$	$[-1.013, 4.97]$	$[-3.651, 4.97]$	$[-3.677, 4.97]$
Car position	[43.7, 83.4]	[40.86, 91.9]	[44.5, 83.4]	[44.5, 88.87]	[44.5, 88.87]

Table 5 provides the quantitative outcomes of this comparison. For simplicity, we present only the projection of the bounds along the variables of interest on a lower dimensional model. As expected, the LGG algorithm performs better in terms of tightness, however unlike our approach, its runtime distinctly increases with the number of iterations. Our implementation of LGG [51] using Convex Polyhedra with octahedral templates becomes slower than the abstractly accelerated version even for small time horizons (our implementation of LGG requires $\sim \!4$ ms for each iteration on a 6-dimensional problem with octahedral templates). Faster implementations of LGG will change the point at which the speed trade-off happens, but abstract acceleration will always be faster for long enough time horizons.

The evident advantage of abstract acceleration is its speed over finite horizons without much loss in precision, and of course the ability to prove properties for unbounded-time horizons.

### Comparison with Alternative Abstract Acceleration Techniques

Table 6 shows a comparison between our approach and [49]. As can be seen, our approach is not only faster but much more precise. The reasons for this are many-fold. In terms of speed, the fact that [49] uses a dynamical model that is double the dimension of the one presented here and that the algorithmic complexity to manipulate it is ${\mathcal {O}}(n^3)$ (*n* being the model dimension) will result in slower computations, even when using sparse matrices. In terms of precision, creating an over-approximation around the centre of the input set, as opposed to the origin, makes a considerable difference, which is increased by the fact that circular over-approximations are contained within the interval hulls used in [49], and are therefore up to $\frac{4}{3}^n$ times smaller in volume. This last consideration is only relevant for rotating dynamics, whereas positive real eigenvalues exhibit the same precision in both approaches. Finally, notice that in the ConvoyCar2 example, where all original eigenvalues are convergent, the interval hull approach [49] creates one divergent eigenvalue, which causes a critical change in the behaviour of the dynamics that leads to unbounded results.

**Table 6:** Comparison on convoyCar2 benchmark [44], between this work and the work in [49]. The intervals give the minimum and maximum values obtained for each variable

Name	Axelerator		Axelerator using [49]	
	100 iterations	Unbounded	100 iterations	Unbounded
Run time	166 ms	166 ms	155085 ms	155085 ms
Car acceleration	$[-0.820, 1.31]$	$[-1.262, 1.31]$	$[-24.24, 23.9]$	$[-\infty , \infty ]$
Car speed	$[-1.013, 5.11]$	$[-4.515, 6.15]$	$[-97.2, 86.7]$	$[-\infty , \infty ]$
Car position	[43.7, 83.4]	[40.86, 91.9]	$[-319, 343.4]$	$[-\infty , \infty ]$

### Scalability

Finally, in terms of scalability, we have an expected ${\mathcal {O}}(p^3)$ complexity with respect to the number of variables *p*, which derives from the simplex algorithm and the matrix multiplications in Eq. (25). We have parameterised the number of cars in the convoyCar example [44] (also seen in Table 3), and experimented with up to 33 cars (each car after the first requires three state variables, so that for example we obtain $(33-1)\times 3= 96$ variables for the 33-car case), using the same initial states for all cars. We report an average obtained from 10 runs for each configuration. These results demonstrate that our method can scale to industrial-size problems (Table 7).

**Table 7:** Scalability features of reachability computations via abstract acceleration

# of variables	3	6	12	24	48	96
Runtime (s)	0.004	0.031	0.062	0.477	5.4	56

## Related Work

There are numerous approaches that tackle the safety verification problem for dynamical models. Time-bounded analysis is unsound in most cases, since it cannot reason about unbounded-time cases (we note that a proof that a given horizon is a completeness threshold [21] would restore such soundness but many tools do not attempt to find such a proof). Unbounded-time solutions are therefore preferred when soundness is required, although they are often either less precise or slower than bounded counterparts.

### Time-Bounded Reachability Analysis

The first approach surrenders exhaustive analysis over the infinite time horizon, and restricts the exploration of model dynamics up to some given finite time bound. Decision procedures for bounded-time reachability problems have made much progress in the past decade, and computational algorithms with related software tools developed. Representatives are STRONG [25], HySon [12], CORA [2], HYLAA [5], Ariadne [7] and SpaceEx [31].

Set-based simulation methods generalise guaranteed integration of ODEs [11, 53] from enclosing intervals to relational domains. They use precise abstractions with low computational cost to over-approximate sets of reachable states up to a given time horizon. Early tools employed polyhedral sets (HyTech [41] and PHAVer [30]), polyhedral flow-pipes [18], ellipsoids [10] and zonotopes [35]. A breakthrough has been achieved by [36, 51], with the representation of convex sets using template polyhedra and support functions. This method is implemented in the tool SpaceEx [31], which can handle dynamical systems with hundreds of variables. It performs computations using floating-point numbers, which is a deliberate choice to boost performance. The downside of this choice is that its implementation is numerically unsound and therefore does not provide genuine formal guarantees, which is at the core of this work. More generally, most tools using eigendecomposition over a large number of variables (more than 10) happen to be numerically unsound owing to the use of unchecked floating-point arithmetic. Some tools (e.g., C2E2 [26]) add a robustness check to the reachability computations in order to restore soundness by over-approximating simulation-based reachability analysis. Another breakthrough in performance has been achieved by HYLAA [5], which is the first tool to solve high-order problems, including several with hundreds of state variables. [7, 17] deal with non-linear dynamics (which are beyond the scope of this work), and the latter does so soundly. Other approaches focus on bounded model checking of hybrid automata, and use specialised constraint solvers (iSAT [27], HySAT [29]), or SMT encodings [19, 39].

### Unbounded Reachability Analysis

The second approach, epitomised by static analysis methods [40], explores unbounded-time horizons. It employs conservative over-approximations to achieve relative completeness (this ensures that we always find a fixpoint) and decidability over infinite time horizons.

Unbounded techniques attempt to infer or compute a *loop invariant*, i.e., an inductive set that includes all reachable states. If the computed invariant is disjoint from the set of bad states, this proves that the latter are unreachable and hence that the loop is safe. However, analysers frequently struggle to obtain an invariant that is precise enough, with acceptable computational costs. The problem is evidently exacerbated by non-determinism in the loop, which corresponds to the case of open systems (i.e., with inputs). Prominent representatives of this analysis approach include Passel [45], Sting [22] and abstract interpreters such as Astrée [8] and InterProc [43].

Early work in this area has used implementations of abstract interpretation and widening [23], which represent still the underpinnings of many modern tools. The work in [40] uses abstract interpretation with convex polyhedra over piecewise differential inclusions. A more recent approach uses a CEGAR loop to improve the precision of polyhedral abstractions [9] following an inductive sequence of half-space interpolants. Dang and Gawlitza [24] employ optimisation-based (max-strategy iteration) with linear templates for hybrid systems with linear dynamics. Relational abstractions [56] use ad-hoc “loop summarisation” of flow relations, while abstract acceleration focuses on linear relations analysis [37, 38], which is common in program analysis.

### Abstract Acceleration

Abstract acceleration [37, 38, 44] captures the effect of an arbitrary number of loop iterations with a single, non-iterative transfer function that is applied to the entry state of the loop (i.e., to the set of initial conditions of the linear dynamics). Abstract acceleration has been extended from its original version to encompass inputs over reactive systems [58] but restricted to subclasses of linear loops, and later to general linear loops but without inputs [44].

The work presented in this article mitigates these limitations by presenting abstract acceleration for *general* linear loops *with* inputs [14, 15], developing numeric techniques for scalability and extending the domain to continuous time models.

The work in [50] puts forward improvements on [14], and its outcomes can be compared with the results in [15, 16]. Indeed, [50] proposes an alternative approach to Abstract Acceleration with inputs, which relies on the expansion of the dynamical equations to a model that is four times the original size (dimension). Although this model is mathematically more tractable, it is slower and more imprecise than the one presented in [15]. The reason for this is that while the latter uses a semi-circular over-approximation of the variable inputs, the former uses a rectangular over-approximation of the dynamics, which is in turn expanded by the acceleration. In particular, this rectangular over-approximation of the dynamics may cause a convergent model to become divergent, which leads to unbounded supports. There are cases in which this rectangular over-approximation can give a tighter result, especially if it is implemented using the symmetry properties described in [15], thus a combined approach may be more efficient. Despite the computational overhead, the handling of the guards presented in [50] has potential advantages over our approach: its description is formal, and indeed our procedure is in general not complete, resulting on occasion in an inability to find reasonably precise bounds. However, in a typical case our procedure works well and in the experiments we have run, it has indeed always been able to find the optimal solution.

## Conclusions and Future Work

We have presented a thorough development of the Abstract Acceleration paradigm to guarded LTI models (namely, conditional linear loops) with inputs. We have extended existing work, which dealt only with autonomous models (i.e., models without inputs). We have decisively shown that the new approach over-competes state-of-the-art tools for unbounded-time reachability analysis in both precision and scalability. In particular, on one hand we claim full soundness (both algorithmic and numerical) of the proposed procedure and of its implementation. On the other hand, the new approach is capable of handling general unbounded-time safety analysis for large-scale open models.

Nested loops are outside of the scope of this contribution, but represent relevant models that ought to be considered. Further work is also needed to extend the approach to non-linear dynamics, which we believe can be explored via hybridisation techniques [3]. We also plan to formalise the framework for general hybrid models, namely dynamical models with multiple discrete locations and location-dependent dynamics under multiple guards.

## Footnotes

1 A more elaborate search of a set of directions might leverage convex optimisation techniques, which come with a computational overhead: as such, they fall outside the scope of this work.

2 As an alternative, a space reduction method can be applied to remove one of the eigenspaces, and the original space can be restored after the reduced reach tube has been evaluated.

3 Note that although we are working in the eigenspace, these sets can be traced back to corresponding sets in the original state space such that $U_b'=\varvec{S}^{-1}BU_b$, $U_c'=\varvec{S}^{-1}BU_c$, and $U_d'=\varvec{S}^{-1}BU_d$. Hence, this inclusion is also valid in the original state space.

4 If *G* is open in a given direction we may pick any number in that direction for the corresponding row of *c*.

5 This is a tighter value than that in [14], where we over-approximated using $n_\theta =\frac{(2\pi )^m}{\prod _j \theta _j}$, where *m* is the number of conjugate pairs.

6 More generally $\left( \varvec{A}-\lambda _s\varvec{I}\right) \varvec{v}_{s,i}=k_{s,i}\varvec{v}_{s,i-1}$, since any vector $k \varvec{v}_{s,i-1}$ is also a generalised eigenvector of $\varvec{A}$, but we select generalised eigenvectors such that $k=1$.

7 http://cps-vo.org/node/30277.

8 http://spaceex.imag.fr.
